# Evolving knowledge graph similarity for supervised learning in complex biomedical domains

**DOI:** 10.1186/s12859-019-3296-1

**Published:** 2020-01-03

**Authors:** Rita T. Sousa, Sara Silva, Catia Pesquita

**Affiliations:** LASIGE, Faculdade de Ciências, Universidade de Lisboa, Lisboa, Portugal

**Keywords:** Knowledge graph, Ontology, Semantic similarity, Machine learning, Genetic programming, Gene ontology, Protein-protein interaction prediction

## Abstract

**Background:**

In recent years, biomedical ontologies have become important for describing existing biological knowledge in the form of knowledge graphs. Data mining approaches that work with knowledge graphs have been proposed, but they are based on vector representations that do not capture the full underlying semantics. An alternative is to use machine learning approaches that explore semantic similarity. However, since ontologies can model multiple perspectives, semantic similarity computations for a given learning task need to be fine-tuned to account for this. Obtaining the best combination of semantic similarity aspects for each learning task is not trivial and typically depends on expert knowledge.

**Results:**

We have developed a novel approach, evoKGsim, that applies Genetic Programming over a set of semantic similarity features, each based on a semantic aspect of the data, to obtain the best combination for a given supervised learning task. The approach was evaluated on several benchmark datasets for protein-protein interaction prediction using the Gene Ontology as the knowledge graph to support semantic similarity, and it outperformed competing strategies, including manually selected combinations of semantic aspects emulating expert knowledge. evoKGsim was also able to learn species-agnostic models with different combinations of species for training and testing, effectively addressing the limitations of predicting protein-protein interactions for species with fewer known interactions.

**Conclusions:**

evoKGsim can overcome one of the limitations in knowledge graph-based semantic similarity applications: the need to expertly select which aspects should be taken into account for a given application. Applying this methodology to protein-protein interaction prediction proved successful, paving the way to broader applications.

## Background

Knowledge discovery in complex domains can be a challenge for data mining methods, which are typically limited to agnostic views of the data, without being able to gain access to its context and meaning. It is widely recognized that the performance of data mining methods can improve significantly when additional relations among the data objects are taken into account, a strategy employed in relational data mining and Inductive Logic Programming [1].

**Fig. 1:**
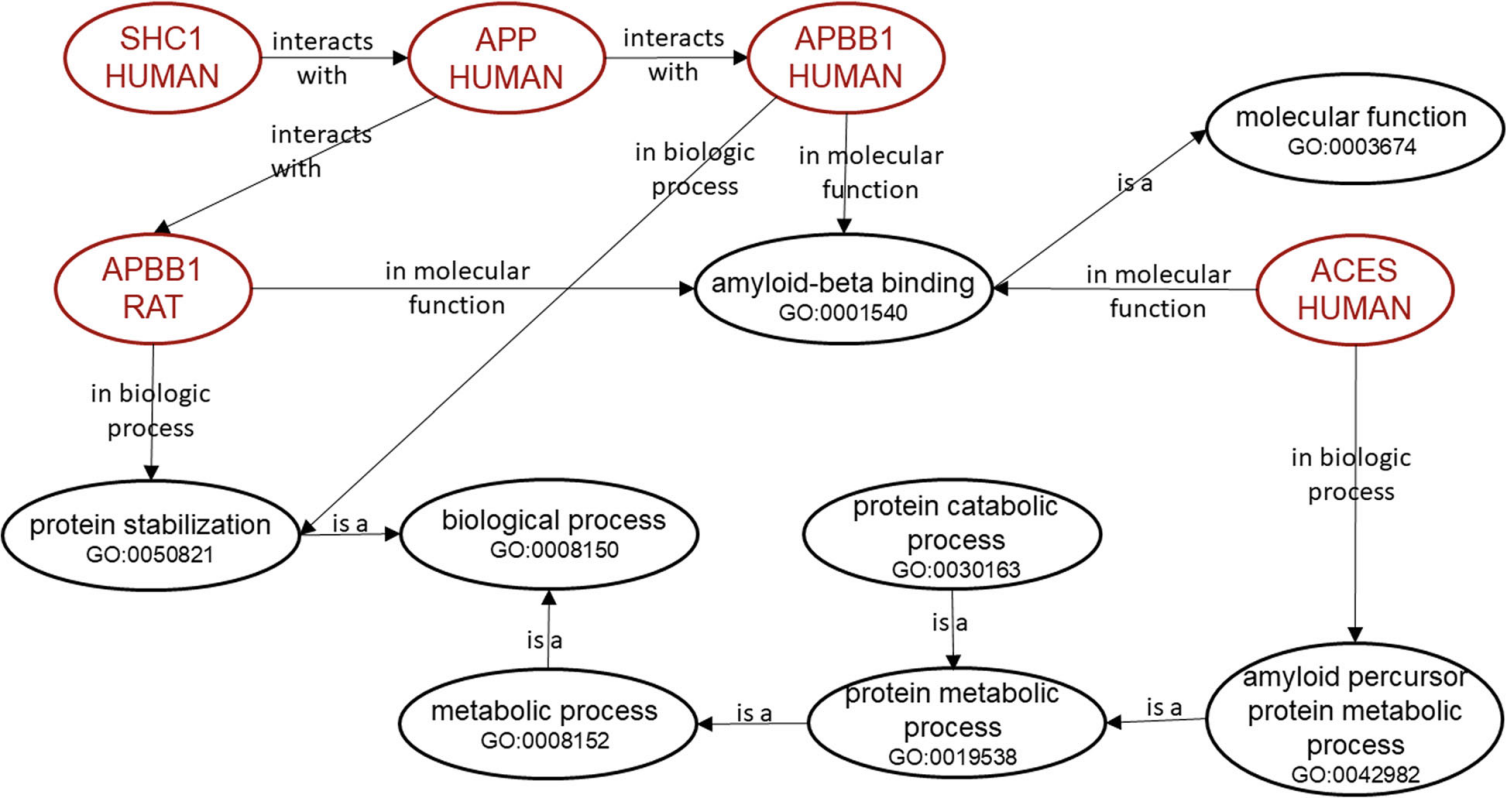
A subgraph of the GO KG illustrating the relationships between proteins. The red nodes are the biological entities (proteins) and the black nodes are the ontology concepts (GO terms)

In the last decade, the explosion in complexity and heterogeneity of biomedical data has motivated a new panorama of semantic data, where millions of semantically-described biological entities are available in knowledge graphs (KGs), through links between ontologies and data [2]. In computer science, an ontology is a formal and explicit specification of a conceptualization in which each term (or concept) is precisely defined and the relationships between terms are parameterized or constrained [3]. Ontologies can be used to represent entities (or instances) in a KG. KGs describe real world entities and their interrelations, through links to ontology concepts describing them, organized in a graph [4]. Gene Ontology (GO) [5] is a very successful biomedical ontology that describes protein function. GO and its associated annotations that link proteins to GO terms make up a KG. Figure 1 shows a small example graph of that KG. Semantic representations of data entities based on KGs that can be explored by data mining approaches provide a unique opportunity to enhance knowledge discovery processes.

In recent years, some approaches combining methods from data mining and knowledge discovery with KGs have been proposed [6]. One of the biggest challenges faced by these approaches is how to transform data coming from KGs into a suitable representation that can be processed by data mining algorithms. Most of the existing approaches build a propositional feature vector representation of the data (i.e., each instance is represented as a vector of features), which allows the subsequent application of most existent data mining algorithms.

The tools FeGeLOD [7] and RapidMiner [8] generate data mining features based on the exploration of specific or generic relations in the graph. Vries et al. [9] use RDF (resource description framework) graph kernels based on intersection graphs and intersection trees to calculate the instances’ feature vectors. More recently, a set of approaches have been developed that can characterize KGs through “embeddings”. In graph embeddings [6], the KG is transformed into sequences of entities, which can be considered as corpus’ sentences. Then, based on the corpus, vector representations are generated using neural language models. Ristoski et al. [10] propose RDF2Vec that uses language modeling approaches for unsupervised feature extraction from sequences of words and adapts them to RDF graphs.

Machine learning approaches that use vectors of features extracted from KGs have also been applied in biomedicine and life science domains. In [11], supervised classifiers predict protein-protein interactions (PPIs) using a set of features to represent a protein pair. In this approach, a protein pair is treated as a bag of words, where the GO terms annotating (i.e., describing) the two proteins represent the words. The feature value of each word is calculated using the concept of information content. Smaili et al. [12] propose Onto2Vec that also uses language modeling approaches to generate vector representations of biological entities in ontologies by combining formal ontology axioms and annotation axioms from the ontology. Onto2Vec is then applied to PPI prediction on different datasets and the identification of protein families. Maetschke et al. [13] use GO-driven algorithms with inducers for protein interaction inference, combining machine learning and KG techniques.

However, the approaches based on vector representations may fail to capture the full underlying semantics. For instance, graph embeddings and graph kernels mostly explore the local structure of KGs. An alternative strategy, and since measuring similarity is fundamental to many machine learning algorithms, is to use the KGs to measure the semantic similarity (SS) [14] between entities in the graph. SS is the computation of the similarity between entities based on their meaning as described in an ontology. For instance, if two biological entities are annotated within the same ontology, we can compare them by comparing the terms with which they are annotated [14].

There are many bioinformatics applications that benefit from using semantic similarity measures (SSMs) over biomedical KGs to compare proteins based on what they do, rather than using sequence similarity, namely: PPI prediction [13, 15–20], prediction of disease-associated genes [15, 21–25], validation of function prediction [26], network prediction [27], prediction of cellular localization [28], and automatic annotation validation [29]. Jain and Bader [17] propose an improved algorithm that uses the SS between GO terms annotated to proteins to distinguish true from false protein interactions. Liu et al. [15] propose a method that incorporates enrichment of GO terms by a gene pair in computing the SS, and apply that method to prediction of sequence homologies, PPIs, and disease-associated genes. Other ontologies have also been used, including the Human Phenotype Ontology [30]. Here, Khöler et al. use SS over phenotypes to diagnose genetic diseases [31], and Hoendorf et al. employ phenotype SS similarity to discover disease related genes [32].

However, a challenge remains. Ontologies aim at modeling a given domain, but within a single domain there can be multiple perspectives, and the SS can be computed taking different aspects into consideration. Let’s take as an example the GO: it describes protein function according to three different perspectives or aspects: biological process, cellular component and molecular function. Therefore, we can compute the SS between two proteins in terms of their annotations within a single aspect, or combining multiple aspects. Different learning tasks may need different perspectives of the KG, and selecting the best aspects or combination of aspects to support a given learning task is not trivial. Usually, the selection of the combination of SS aspects is based on a researchers’ intuition and experience. For instance, if the learning task is the prediction of interaction between proteins, it is expected that similarity in biological process or cellular component are stronger indicators for protein interaction than similarity in molecular function. Therefore, a combination in which biological process and cellular component aspects have more weight will probably be the choice of researchers. Both Jain and Bader [17] and Maetschke et al. [13] have found this to be true.

However, not all tasks have such a clear choice of combination. For instance, if the learning task is the prediction of disease-associated genes, how to combine molecular function with the remaining two aspects is not straightforward. Automating the selection of the best combination of KG aspects to support specific tasks would simplify and generalize the application of these techniques, rendering it more independent of expert knowledge.

In this work, we propose a novel methodology, evoKGsim, that uses Genetic Programming (GP) [33] over a set of semantic similarities, each computed over a different semantic aspect of the underlying data, to arrive at the best combination between the different aspects to support different supervised learning tasks. GP is chosen for its ability to search large solution spaces by means of evolving a population of free-form readable models through crossover and mutation. Unlike most search and optimization methods, which try to optimize the values of variables, GP tries to optimize a combination of variables and operators/functions, which is suitable for finding the best combinations of semantic similarity scores. This methodology is applied to PPI prediction and evaluated in benchmark datasets. We focus on this problem since the relationships between the different semantic aspects and potential classification performance are well established.

## Results

A key aspect of our evaluation approach is to compare evoKGsim, that is able to evolve a combination of semantic aspects, to static combinations established a priori. This allows us to compare our methodology to a scenario where semantic aspects are selected and combined by experts before the prediction task. We have used five static combinations as baselines: the biological process (BP), molecular function (MF), and cellular component (CC) single aspects, and the average (Avg) and maximum (Max) of the single aspect scores. Furthermore, we also compare evoKGsim to combinations selected by an exhaustive search method and decision tree models.

To establish the performance of the static baselines, the prediction of PPI is formulated as a classification problem where a SS score for a protein pair exceeding a certain threshold (SS cutoff) indicates a positive interaction. The SS threshold is chosen after evaluating the weighted average of F-measures (WAF) at different threshold intervals and selecting the maximum. This emulates the best choice that a human expert could theoretically select.

Regarding exhaustive search combinations, we performed a grid search approach over the weights of each semantic aspect as well as the threshold for classification, where weights were used in a linear combination.

To provide a comparison of our methodology results against the results of another classification method not based on evolutionary algorithms, we employed decision trees using the SS of the three semantic aspects as input features.

By comparing the performance of these alternative approaches to the performance of evoKGsim, we aim at investigating the ability of GP to learn combinations of semantic aspects that are able to support improved classification performance.

### Static combinations

Prior to performing the comparative evaluation, we investigated the behavior of the different SS approaches employed, coupled with the different baselines.

**Fig. 2:**
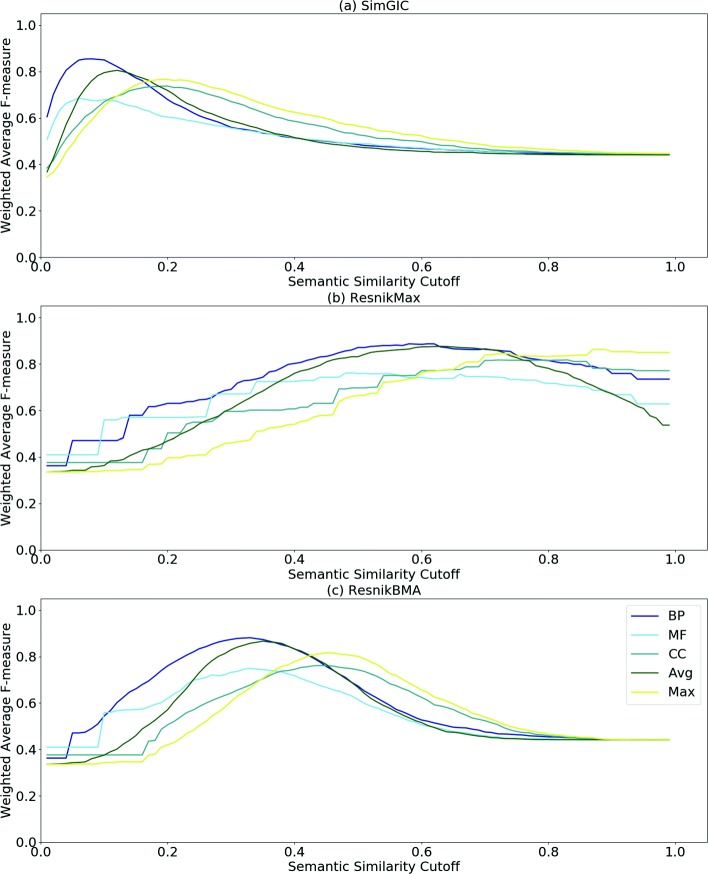
WAF Curves for DIP-HS PPI dataset. WAF evaluations with static combinations of semantic aspects (CC, BP, MF, Avg and Max) at different cutoffs are shown. The evaluation is performed using three SSMs: **a** SimGIC, **b** Resnik_Max_ and **c** Resnik_BMA_

**Fig. 3:**
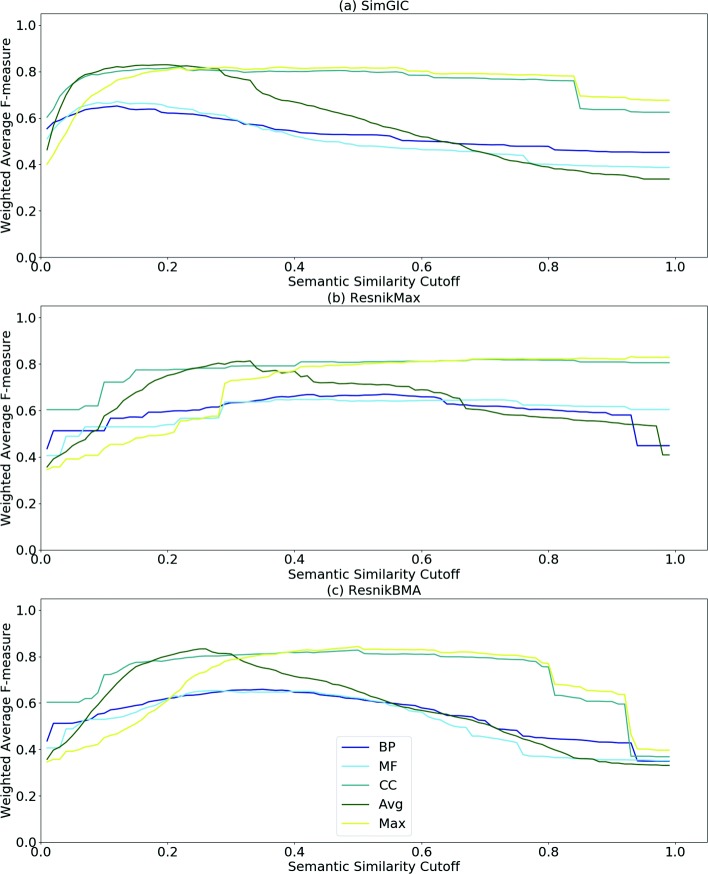
WAF Curves for STRING-EC PPI dataset. WAF evaluations with static combinations of semantic aspects (CC, BP, MF, Avg and Max) at different cutoffs are shown. The evaluation is performed using three SSMs: **a** SimGIC, **b** Resnik_Max_ and **c** Resnik_BMA_

Figures 2 and 3 show the WAF of classification at different cutoffs with three SSMs for the DIP-HS and STRING-EC PPI datasets, respectively. While Fig. 2 is representative of the behavior found for the other datasets, Fig. 3 shows a different behavior, where the F-measure is less penalized at higher cutoffs, particularly for the Max and CC results. The proteins in this dataset have fewer BP annotations, which may help explain the improved performance of CC. Additional file 1 shows the results for the remaining datasets.

Comparing the charts for different SSMs, we observe that, for each set of curves, the maximum F-measure is achieved at different ranges of SS cutoff. For SimGIC (Fig. 2a), Resnik_Max_ (Fig. 2b) and Resnik_BMA_ (Fig. 2c) the ranges are approximately [0.1−0.3], [0.6−0.8] and [0.3−0.5], respectively. For most datasets, each SSM shows a consistent behavior with curves having similar shapes. Furthermore, we verify that the maximum observed F-measure is achieved when Resnik_Max_ is used.

Static combinations were evaluated using stratified 10-fold cross-validation. The training set is used to select the best classification threshold which is then applied to the test set. Table 1 presents the median WAF achieved in each baseline.

**Table 1:** Median of WAFs with alternative methodologies and with evoKGsim for the different PPI datasets

Dataset	SSM	Single and static combinations					Exhaustive search	Decision	evoKGsim
(#interactions)		BP	CC	MF	Avg	Max	Combinations	Trees	
STRING-EC	SimGIC	0.648	0.822	0.670	0.825	0.814	0.825	0.804	**0.826**
(2245)	Resnik_Max_	0.670	0.819	0.641	0.806	0.826	0.817	**0.884**	0.864
	Resnik_BMA_	0.661	0.828	0.642	0.831	0.848	0.832	0.837	**0.849**
STRING-DM	SimGIC	0.891	0.880	0.791	0.890	0.891	0.927	0.855	**0.936**
(550)	Resnik_Max_	0.910	0.899	0.799	0.927	0.927	0.936	0.917	**0.937**
	Resnik_BMA_	0.928	0.871	0.794	0.936	0.918	**0.963**	0.927	0.945
BIND-SC	SimGIC	0.849	0.831	0.715	0.854	0.840	0.868	0.830	**0.876**
(1366)	Resnik_Max_	0.883	0.845	0.775	0.904	0.908	**0.923**	0.890	**0.923**
	Resnik_BMA_	0.864	0.842	0.754	0.901	0.868	**0.908**	0.872	0.901
DIP/MIPS-SC	SimGIC	0.811	0.776	0.690	0.803	0.779	0.818	0.754	**0.825**
(13807)	Resnik_Max_	0.845	0.798	0.703	0.835	0.838	**0.854**	0.840	0.849
	Resnik_BMA_	0.820	0.788	0.698	0.835	0.822	0.842	0.780	**0.843**
STRING-SC	SimGIC	0.802	0.764	0.684	0.804	0.780	0.814	0.766	**0.817**
(30384)	Resnik_Max_	0.825	0.788	0.682	0.834	0.826	0.839	**0.843**	**0.843**
	Resnik_BMA_	0.818	0.784	0.678	0.837	0.817	0.837	0.793	**0.838**
DIP-HS	SimGIC	0.840	0.746	0.698	0.823	0.768	0.857	0.799	**0.861**
(2739)	Resnik_Max_	0.892	0.829	0.770	0.885	0.867	**0.914**	0.894	0.894
	Resnik_BMA_	0.874	0.773	0.754	0.876	0.811	0.872	0.867	**0.881**
STRING-HS	SimGIC	0.824	0.769	0.700	0.813	0.786	0.823	0.774	**0.830**
(6912)	Resnik_Max_	0.848	0.763	0.723	0.850	0.811	**0.868**	0.850	0.867
	Resnik_BMA_	0.851	0.792	0.725	0.861	0.815	0.870	0.816	**0.876**
GRID/HPRD-unbal-HS	SimGIC	0.686	0.652	0.621	0.685	0.664	**0.701**	0.621	0.694
(31320)	Resnik_Max_	0.718	0.674	0.655	0.729	0.702	**0.734**	0.703	**0.734**
	Resnik_BMA_	0.717	0.678	0.646	0.737	0.697	**0.742**	0.662	**0.742**
GRID/HPRD-bal-HS	SimGIC	0.647	0.630	0.618	0.672	0.647	**0.674**	0.590	0.673
(31349)	Resnik_Max_	0.656	0.602	0.590	0.648	0.636	**0.664**	0.636	0.654
	Resnik_BMA_	0.652	0.640	0.597	0.673	0.659	0.674	0.604	**0.677**

In bold, the best result for each dataset-SSM pair. The median WAF achieved for each baseline is underlined when evoKGsim significantly outperforms the baseline (using *α*=0.01)

### Exhaustive search combinations and decision tree models

The exhaustive search method is based on a grid search over a set of possible values for the SS threshold (values in the range from 0 to 1 with a step of 0.05) and a set of possible values for SS score weights (values in the range from 0 to 1 with a step of 0.1), using the WAF of classification on training set as the optimization criterion. The components of the candidate solutions are then a SS threshold and three weights used to calculate the weighted average of the three SS scores. The number of potential solutions was established to be roughly equal to the number of candidate solutions evaluated by GP.

The decision tree models were obtained using the Decision Tree package of scikit-learn 0.20.2 [34] with default parameters.

Exhaustive search combinations and decision tree models were evaluated using 10-fold cross-validation. The median WAF for all datasets is presented in Table 1.

### Comparative evaluation

Table 1 shows the median WAF of stratified 10-fold cross-validation for the static combinations, the exhaustive search combinations, the decision tree models and evoKGsim, using different SSMs.

The statistical significance of the experimental results was determined using pairwise non-parametric Kruskal-Wallis tests [35] at *p*< 0.01. All statistical analyses were performed using the Python library SciPy 1.3.1 [36]. Table S3 of Additional file 1 shows the *p*-values for the Kruskal-Wallis test for comparisons between evoKGsim and all the other alternative methodologies over the nine PPI datasets. In Table 1, for each dataset-SSM pair, the median WAF achieved for each alternative methodology is underlined when the performance differences between evoKGsim and that methodology are statistically significant.

### evoKGsim for intra-species prediction

**Fig. 4:**
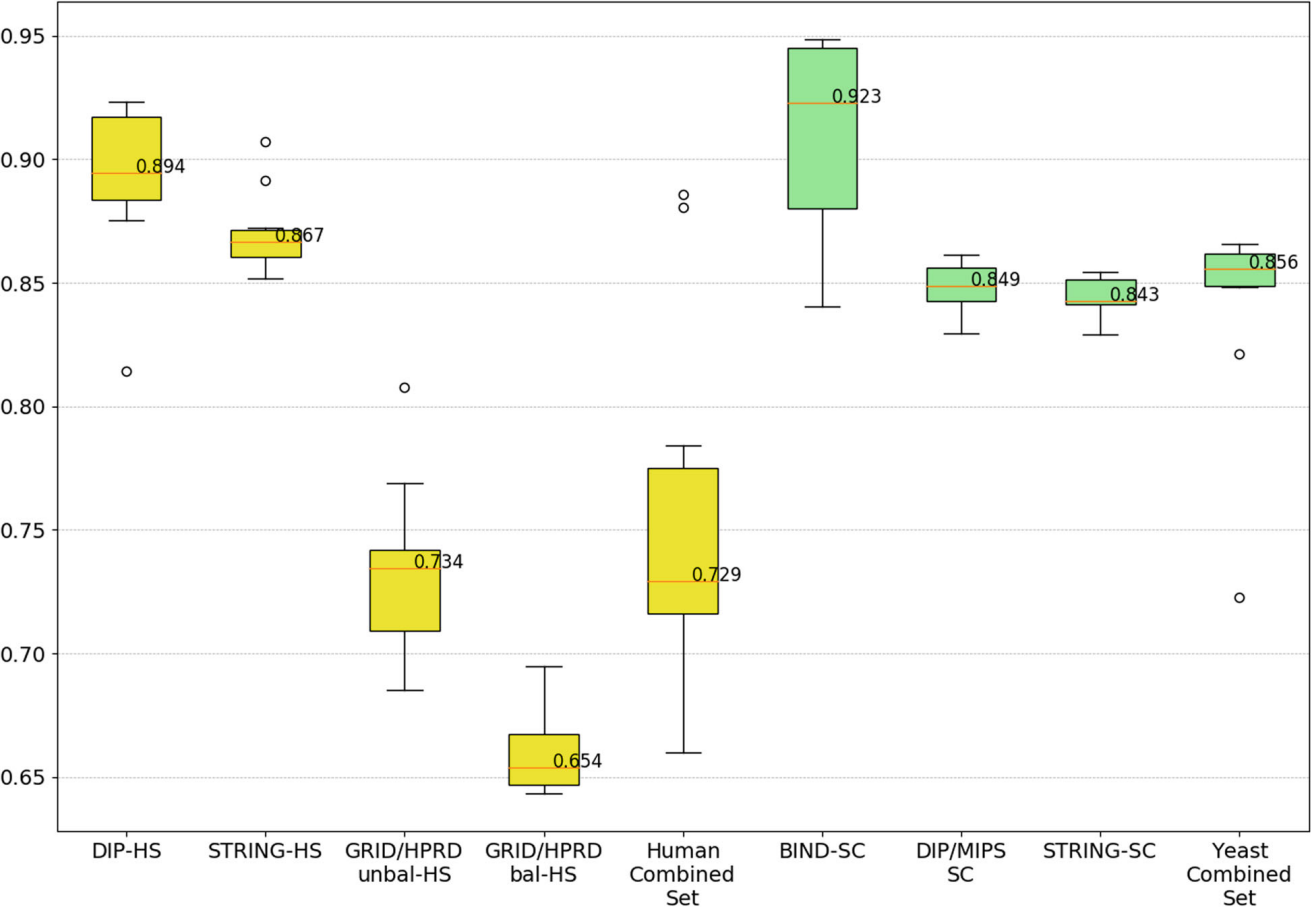
WAF Boxplot using combined sets. The yellow boxes represent the WAF of predictions for human data and the green boxes represent the WAF of predictions for yeast data. Within the same species, the datasets appear on the x-axis in ascending order of size. The median of the WAF values is indicated by the bar within a box

The previous results suggest that having fewer instances can hinder the ability of GP to learn a suitable combination of aspects. Therefore, and since two of the species have several datasets, we tested evoKGsim using combined sets for each of these species. This allows us to investigate whether a species-oriented model based on more instances can improve on the performance of individual datasets. The human combined set contains the data from 4 datasets (STRING-HS, DIP-HS, GRID/HPRD-bal-HS, GRID/HPRD-unbal-HS), with a total of 54219 protein pairs. The yeast combined set contains the data from three datasets (STRING-SC, BIND-SC, and DIP/MIPS-SC), with a total of 42330 protein pairs. Some pairs of proteins appear in more than one dataset so, in these combined sets, the repeated pairs are first removed from the combined sets and only then randomly split into training and test sets. Figure 4 shows the WAF boxplot for the three yeast datasets, the four human datasets, the yeast combined set and the human combined set. Each box includes the WAFs obtained in 10-fold cross-validation.

Using the boxplots to compare the prediction performance, we conclude that, for both species, the performance using the combined set is similar to the performance of the larger datasets included in that combined set. This may be explained by the influence of the large proportion of instances coming from the larger datasets, such as GRID/HPRD-unbal-HS and GRID/HPRD-bal-HS for human and STRING-SC for yeast, although for human this influence is less pronounced.

We were also interested in investigating, within a species, the performance of training in a given group of datasets and testing on a different one. Once again, to solve the problem of repeated pairs, we determine that if a protein pair is simultaneously in the training set and in the test set, it will be removed from one of them. Tables 2 and 3 present the different tests we conducted, indicating for each test which datasets are in the training set and which are in the test set for human and yeast data, respectively. This strategy does not support stratified cross-validation so results are based on 10 independent runs.

**Table 2:** Training and test sets and number of protein pairs respectively used in each experiment

Training set	No. of pairs	Test set	No. of pairs
S	6912	D+Gub+Gb	47307
D	2739	S+Gub+Gb	51480
Gb	31349	D+S+Gub	22870
Gub	31320	D+S+Gb	22899
S+Gb+Gub	69581	D	2115
D+Gb+Gub	65408	S	5037
S+D	9651	Gb+Gub	44929
Gb+Gub	62669	S+D	7239
S+Gb	38261	D+Gub	17746
D+Gub	34059	S+Gb	20697
S+Gub	38232	D+Gb	17771
D+Gb	34088	S+Gub	20668

The names of the datasets STRING-HS, DIP-HS, GRID/HPRD-unbal-HS, and GRID/HPRD-bal-HS are abbreviated to “S”, “D”, “Gub” and “Gb”, respectively

**Table 3:** Training and test sets and number of protein pairs respectively used in each experiment

Training set	No. of pairs	Test set	No. of pairs
S	30384	B+D	11946
D	13807	S+B	28523
B	1366	S+D	40964
S+B	31750	D	11163
S+D	44191	B	713
B+D	15173	S	27639

The names of the datasets STRING-SC, BIND-SC, and DIP/MIPS-SC are abbreviated to “S”, “B”, and “D”, respectively

**Fig. 5:**
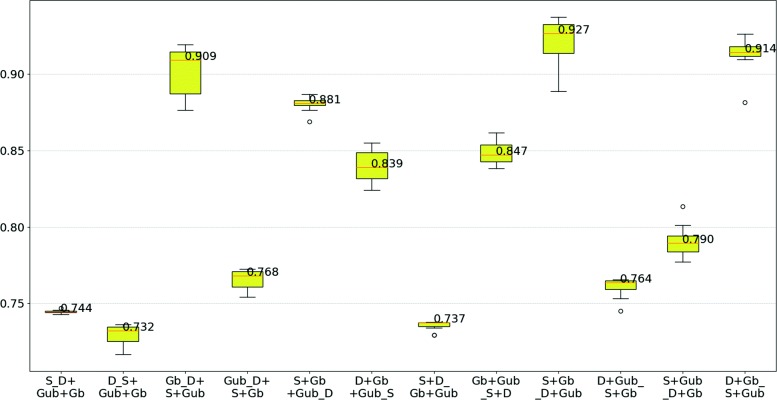
WAF Boxplot using human datasets to training and testing. The labels of the plots are in format ’D1+D2_D3+D4’, where D1, D2, D3, D4 are the original datasets, D1+D2 is the training set that contains data from D1 and D2, and D3+D4 is the test set that contains data from D3 and D4. In the labels, the names of the datasets STRING-HS, DIP-HS, GRID/HPRD-unbal-HS, and GRID/HPRD-bal-HS are abbreviated to “S”, “D”, “Gub”, and “Gb”, respectively

**Fig. 6:**
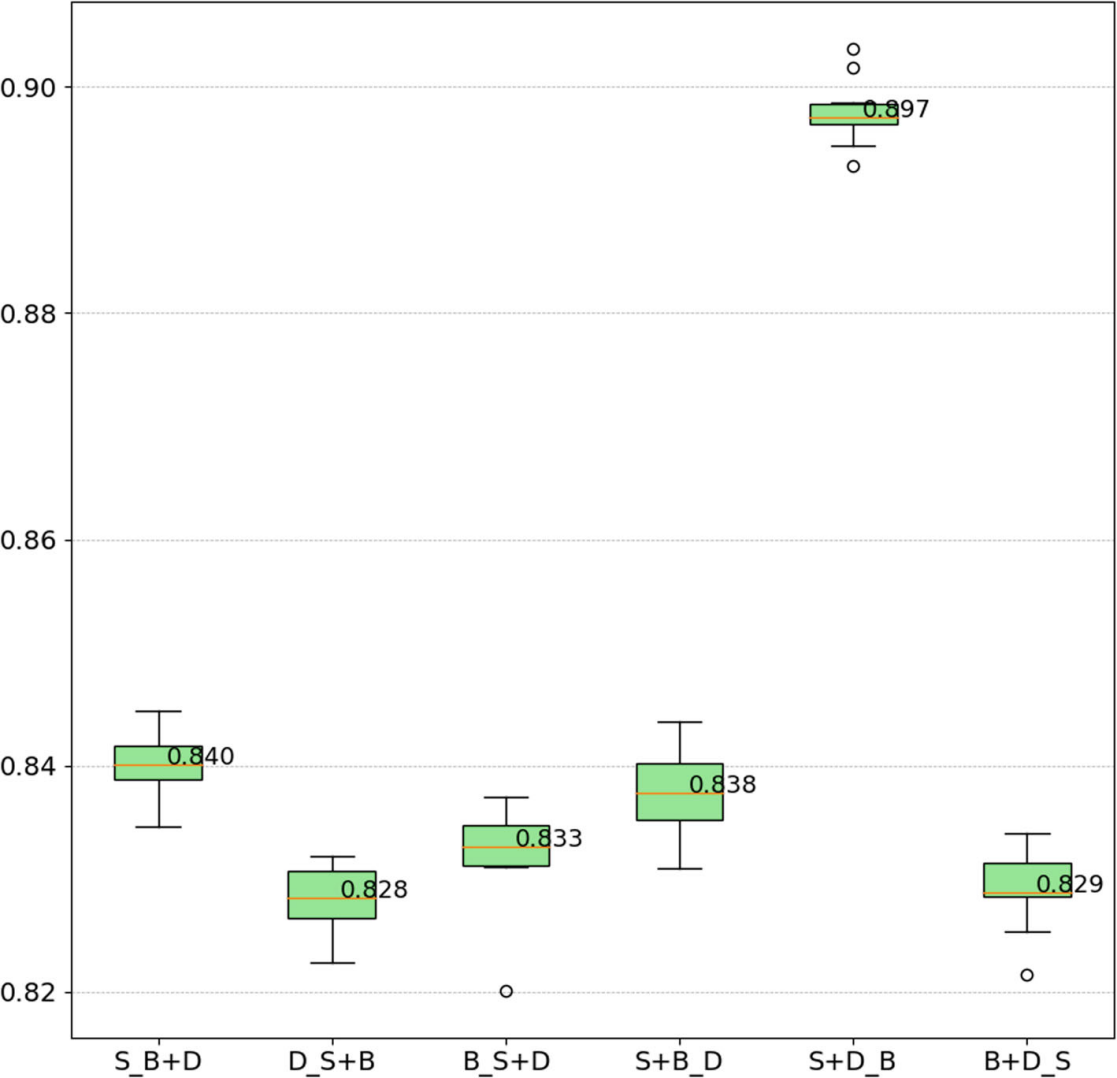
WAF Boxplot using yeast datasets to training and testing. The labels of the plots are in format ’D1+D2_D3+D4’, where D1, D2, D3, D4 are the original datasets, D1+D2 is the training set that contains data from D1 and D2, and D3+D4 is the test set that contains data from D3 and D4. In the labels, the names of the datasets STRING-SC, BIND-SC, and DIP/MIPS-SC are abbreviated to “S”, “B”, and “D”, respectively

The results for human and yeast are summarized in Figs. 5 and 6, respectively. Analyzing the results for human sets, we conclude that using a larger dataset for training can improve the performance of classification. For instance, training with data from GRID/HPRD-bal-HS (e.g., S+Gb_D+Gub), the larger dataset, leads to higher test WAFs, while training with fewer data points (e.g., D_S+Gub+Gb) leads to lower WAF values. Relatively to yeast sets, the same behavior is observed. For instance, in S+D_B the experiment with the largest training set and the smallest test set, WAF is more than 5% higher than in the second best performing case.

### evoKGsim for cross-species prediction

In the above analysis, the training and test data come from the same species. However, training prediction methods on one species’ data and testing them on another species’ protein pairs may be useful to explore, since GO annotation is designed to be species independent [5].

**Fig. 7:**
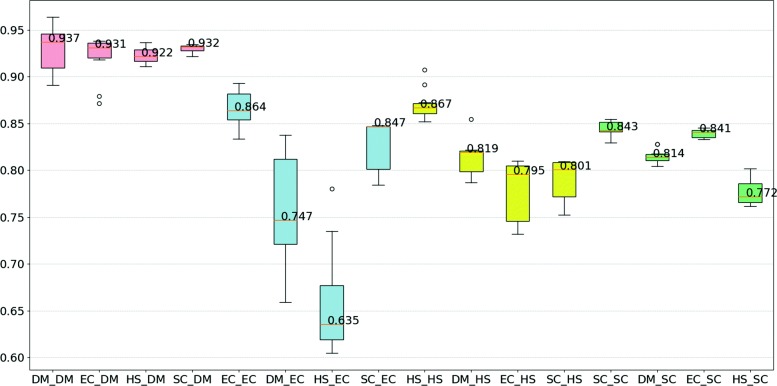
WAF Boxplot using one species to train and another species to test. ’D1_D2’ format of the labels means training with D1 and testing on D2

To test this idea, we use evoKGsim to predict PPI but, using one species’ data to train the model and another species’ data to test it. Figure 7 displays the self-test WAF boxplot (obtained using 10-fold cross-validation) and cross-species-test WAF boxplot (obtained in 10 independent runs) using four datasets (STRING-DM, STRING-EC, STRING-HS, STRING-SC) of four different species.

The results reveal that evoKGsim is generally more effective when trained and tested using data from the same species than when trained with data from one species and tested with data from another species. For *D. melanogaster*, performances are very similar across training sets. For *E. coli*, performance can differ greatly, with the human training set decreasing performance by more than 27% when compared to *E. coli*.

### evoKGsim for multi-species prediction

We also tested evoKGsim by training the model using all species data except the one species that was used for testing and performing 10 runs. Additionally, we also ran a species-agnostic 10-fold cross-validation experiment where the data from all datasets was combined into a single dataset. The strategy to remove repeated pairs used before in evolved combinations species-oriented is applied.

**Fig. 8:**
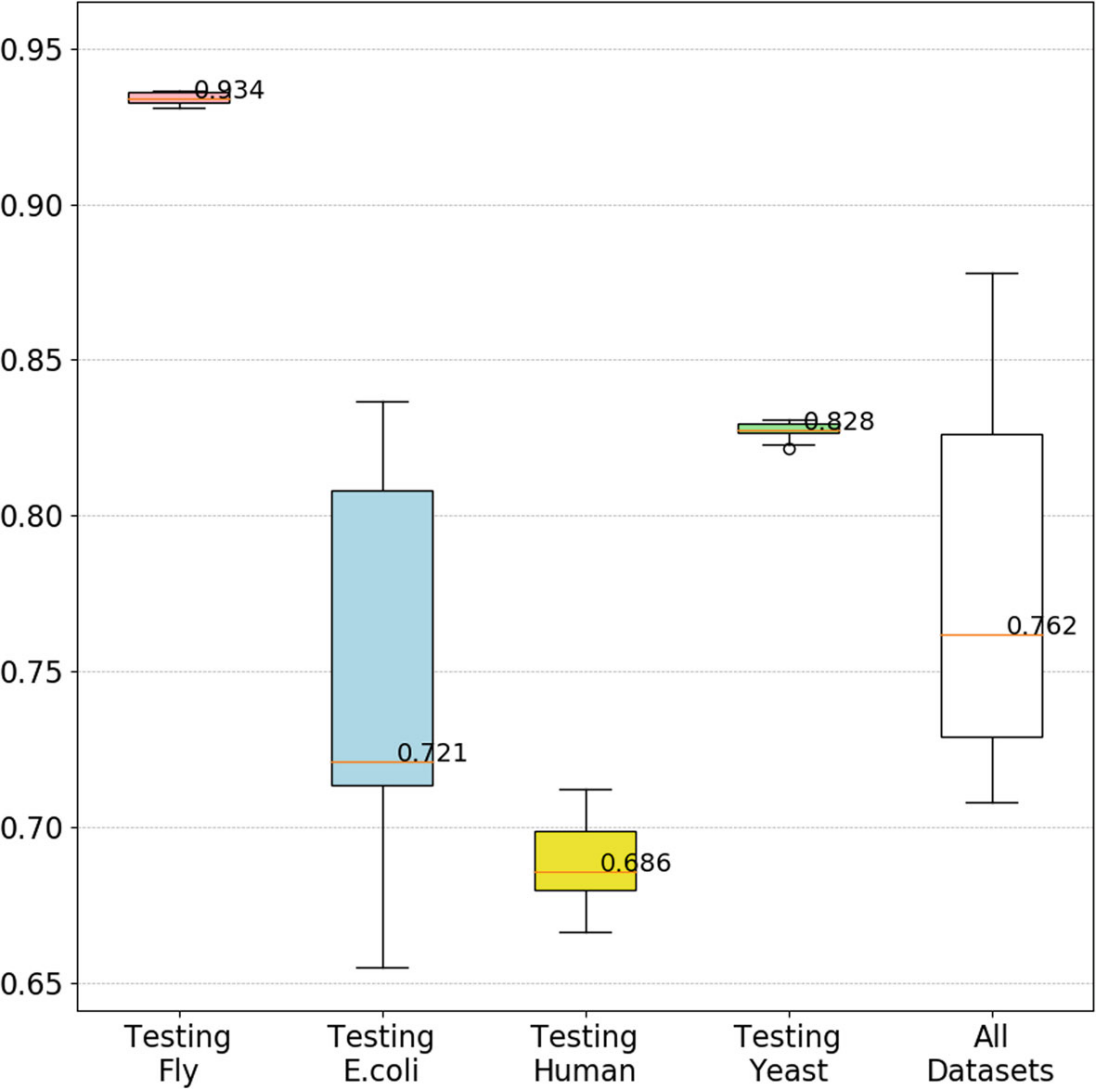
WAF Boxplot using multispecies data in training set

In Fig. 8 we can observe some interesting effects. For *D. melanogaster* and *S. cerevisiae*, the differences observed between training with the other species or with the same species are rather small: *D. melanogaster* multiple species performance decreases by 0.3%, whereas for *S. cerevisiae* it decreases by 3.3%. However, for *E. coli* and human, the difference is more substancial, with *E. coli* dropping performance by 16.6% and human by 5.9%. Interestingly, the experiment that uses the data from all the datasets produced a mid-range WAF value, indicating that it is possible to produce a successful species-agnostic model.

### Overview of GP models

Since GP produces potentially readable models, after evaluating the performance of evoKGsim, the models generated by GP across different datasets are analyzed. The goal is to identify which are the operators and combinations that GP uses more often, and how they compare across datasets. The analysis of the models is conducted using the Python library SymPy 1.3 [39] and the Python package Graphviz 0.10.1 [40]. Table 4 summarizes, for the 10 folds performed in each dataset, the average length (number of tree nodes) of the models and the average relative frequency of variables BP, CC and MF in the models. These are calculated after arithmetic simplification (using SymPy) of the best solutions returned by GP, that is applied to remove redundant code.

**Table 4:** Analysis of GP models for each dataset

Dataset	BP	CC	MF	Length
STRING-EC	0.362	0.446	0.192	66.9
STRING-DM	0.401	0.337	0.263	134.8
BIND-SC	0.327	0.397	0.277	128.9
DIP/MIPS-SC	0.479	0.359	0.162	30.9
STRING-SC	0.404	0.387	0.209	52.5
DIP-HS	0.477	0.337	0.187	61.7
STRING-HS	0.434	0.306	0.260	35.7
GRID/HPRD-unbal-HS	0.371	0.337	0.292	30.3
GRID/HPRD-bal-HS	0.452	0.289	0.259	36.5
Average	0.412	0.355	0.233	64.244
Species-agnostic	0.473	0.390	0.137	55.1

As expected, variable MF appears less frequently in the GP models. These results are in agreement with the previous results that indicated that BP and CC annotations are stronger indicators for PPI than MF annotation. However, the frequency in which a given variable appears in a GP model does not necessarily measure its importance for the predictions, as its effect may be stronger or weaker depending on its surrounding context. The average length of the GP models is 64.2, with somewhat large differences between datasets. One interesting observation is that, when the datasets are smaller, such as STRING-DM and BIND-SC, the average length of the GP models tends to increase. This may be an indication that GP is evolving highly tuned, possibly overfitted models, for lack of sufficient data to induce smaller and more general ones. However, in GP the complexity of a model does not depend on its size, but on the particular features and operators used to build it, and therefore one cannot assume that larger models overfit more than smaller ones [41].

In GP models of the species-agnostic experiment the differences between the frequencies of the variables BP, CC and MF are more substancial, being MF the least frequent variable and BP, clearly, the most frequent variable (last row of Table 4). Once again the results indicate that similarities in BP and CC annotations are stronger indicators for PPI than MF annotation, with a slight advantage for BP.

## Discussion

### Comparison with static combinations

For all datasets, GP is able to learn combinations of semantic aspects that improve the best classification performance obtained by the static baselines for that dataset.

Regarding static combinations approaches, the differences between SSMs are not unexpected since SimGIC considers multiple GO annotations for calculating SS while Resnik approaches only consider the best-matching term pairs. Therefore, the better performance using Resnik_Max_ makes sense because proteins in PPIs only need to be in proximity in a single location or participate in a single shared biological process, to be biologically relevant for PPI prediction. As expected, the results indicate that the predictive power of the BP and CC aspects is similar, with a slight advantage for BP, while the predictive power of MF is considerably lower. The dataset STRING-EC (Fig. 3) is an exception because using only the SS for BP ontology provides worse results comparatively to the other combinations of single aspects. Once again, the explanation for that can be the lack of BP annotations for the species *E. coli*. The Avg combination outperforms the Max in most cases. This is possibly due to the fact that the Avg combination can take into consideration both the BP and the CC aspects.

Regarding evoKGsim, improvements over the single aspect baselines are, as expected, more pronounced for MF (up to 26%) than for the other aspects. The improvements are also clear when considering the combination baselines (2-7% in most cases). evoKGsim significantly outperforms the MF baseline in any dataset with any SSM. In accordance with static combinations results, the importance of MF to predict PPI is also reduced in evoKGsim as is evidenced by its lower frequency in the GP models. For the remaining static baselines, in all dataset-SSM pairs, except the GRID/HPRD-bal-HS - Resnik_Max_ pair, the performance of evoKGsim is always slightly better than the static baselines, but sometimes not enough to be statistically significant.

It is important to note that the baselines were built to emulate the scenario of a researcher choosing an optimal threshold and employing two well-known strategies for combining the single aspect scores. With GP, we have always used the 0.5 cutoff with no further tuning, and have used a function set that included the maximum but not the average (which interestingly did not guarantee success or failure when compared to these two baselines). It is interesting to note as well, that often evoKGsim achieves its best WAF when used with Resnik_Max_ (in five out of nine datasets). Resnik_Max_ is also the best overall measure for the single aspect baselines. For that reason, in the experiments in sections dedicated to intra-, cross-, multi-species prediction and overview of GP models, the results are obtained using only Resnik_Max_ as SSM.

### Comparison with exhaustive search combinations

In four out of nine datasets, evoKGsim performs better than combinations selected by exhaustive search, and achieves the same performance in two datasets. However, the statistical tests reveal that, in the majority of cases, evoKGsim is not able to significantly outperform the exhaustive search approach. Nevertheless, when evoKGsim has a worse performance, these differences are never statistically significant.

**Fig. 9:**
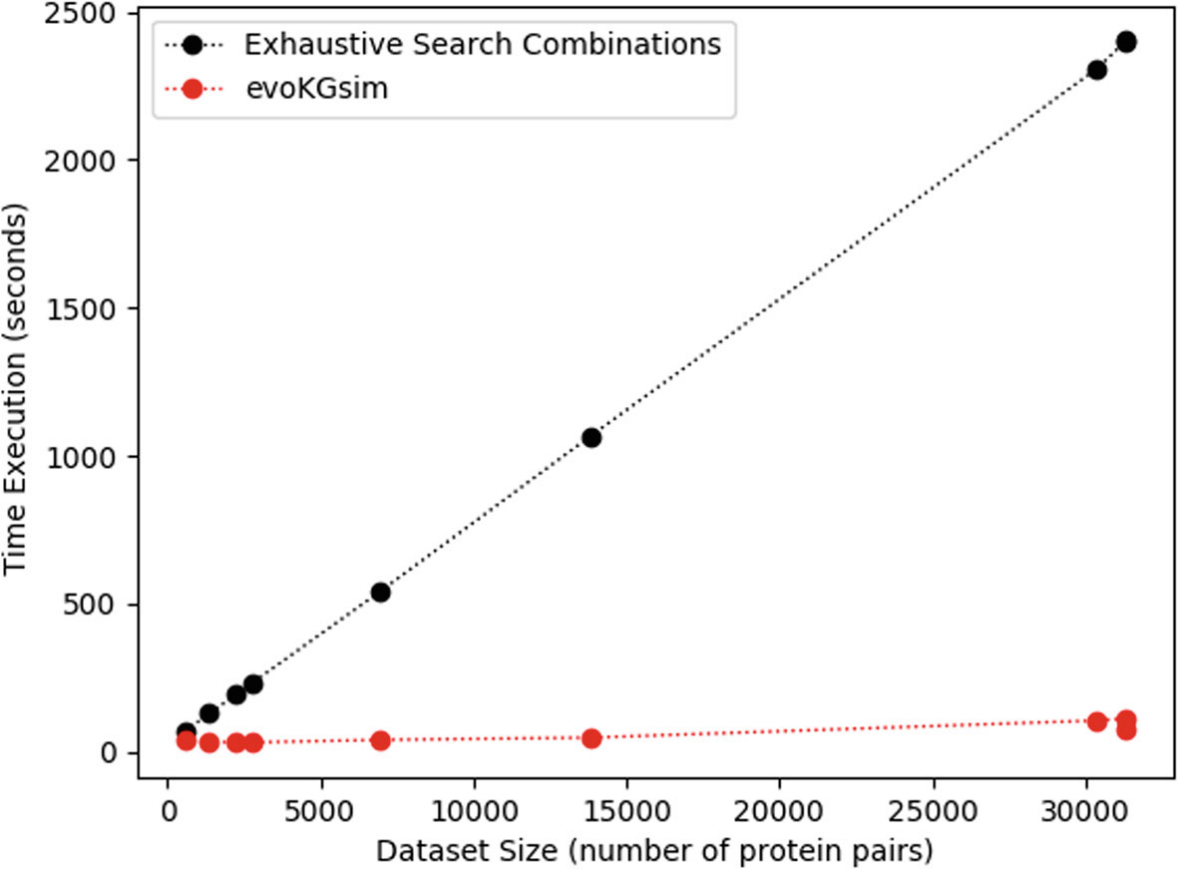
Plot of median execution time versus dataset size

It also should be taken into account that 20,000 parameter combinations are tested in search of the combination of SS weights and SS threshold that maximizes the WAF of PPI prediction. In opposition, evoKGsim is based on a genetic algorithm that explores, in an efficient way, the space of possible solutions to obtain the combination of SS scores that maximizes the WAF of the classification. To investigate differences in computational performances, we compared the training and testing times of exhaustive search combinations and our methodology. To visualize these results, Fig. 9 shows the variation of the median execution time with the size of the dataset for each methodology (exhaustive search combinations and evoKGsim). We observe that evoKGsim is not only faster, but also more scalable than the exhaustive search method. Although training and testing times depend on the implementation, there are such large differences in times that the differences cannot be attributed only to implementation.

### Comparison with decision tree models

In eight out of nine datasets, evoKGsim is able to learn combinations of semantic aspects that improve the best classification performance obtained by decision trees. These differences are statistically significant in six cases. The only dataset where evoKGsim is unable to improve the performance (STRING-EC) is one of the smallest (<2500 protein pairs), which may help explain the lower performance of our approach. For this dataset, we achieve 2.3% lower performance, but this difference is not statistically significant. Furthermore, we verified that the obtained decision tree models are too large for human understanding in nearly all cases, producing models with hundreds of leaves.

### Comparison of species-based aggregation of data

**Fig. 10:**
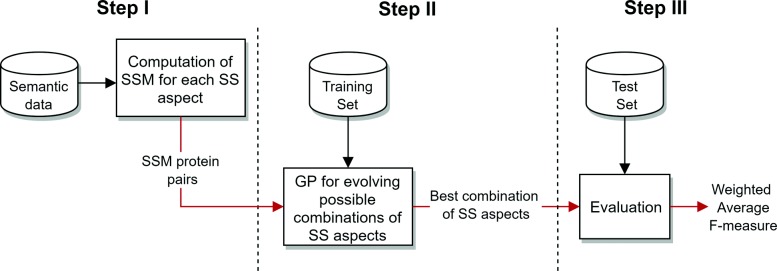
Overview of the evoKGsim methodology

**Fig. 11:**
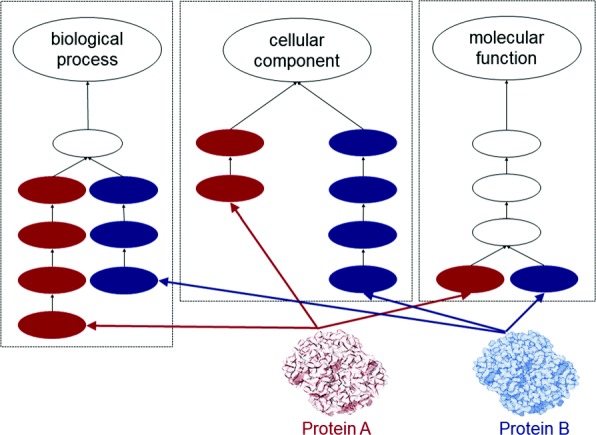
Illustration of a directed acyclic graph representing GO terms annotating two proteins. Red terms annotate only protein A, blue terms annotate only protein B and white terms annotate both proteins A and B

Our results suggest that having fewer instances can hinder the ability of GP to learn a suitable combination of aspects. This motivated different strategies for aggregating datasets based on species. Regarding predictions based on different combinations of datasets within the same species (see Figs. 10 and 11 and Table 1), we verify that prediction methods are always more effective when trained and tested with the same dataset than when trained with other datasets of the same species. This is not surprising, considering how easy it is for biases to be unintentionally included in a dataset, and how much of these biases can be captured and used by a powerful method like GP, as long as they help achieve a good performance. Potential sources of bias could be a direct result of the scientific process, where determining the interaction of proteins is likely to target proteins that are more abundant [42] or that participate in relevant processes, e.g. resistance/susceptibility to disease or stress conditions.

Regarding cross-species prediction, evoKGsim is generally more effective when trained and tested using data from the same species. In fact, training with human data gives consistently the worst results. This could be a result of the human dataset being composed of proteins that bear a lower similarity to those in other species datasets or of differences in the annotation process.

Park [43] and Maetshke et al. [13] also evaluated the cross-species accuracy by training a sequence-based classifier on one species data and predicting interactions for another species. Park found that datasets typically used for training prediction methods contain peculiar biases that limit the general applicability of prediction methods trained with them. In strong contrast, Maetshke et al. conclude that datasets linked to low self-test accuracy result in low cross-species accuracies while datasets with high self-test accuracy indicate datasets of good quality and, consequently, lead to high test accuracies for all training sets. This means that, according to Maetshke et al., the prediction performance on the test species for different training species largely depends on the self-test accuracy achieved on the test dataset and only to a lesser degree on the training dataset. Interestingly, the results for evoKGsim do not seem to indicate that datasets with high self-test WAF (such as STRING-DM) lead to high test WAF for all training sets.

Finally and considering the use of diverse training data will likely produce more generally applicable models, we also investigated applying a model learnt from more than one species data to the classification of another species data. This yielded interesting results with a successful creation of a species-agnostic model.

### Other PPI prediction methods

By using benchmark datasets, our results could be in principle directly compared to the results obtained by other works using the same datasets. However, our results cannot be directly compared to the published ones, first because we used more recent versions of the GO KG, and second because we needed to exclude some protein pairs of the benchmark datasets. The results obtained in different works are also not directly comparable between themselves. Nevertheless, the results from relevant related work were compiled, to support a comparative overview.

Table 5 summarizes the area under the receiver operating characteristic curve (AUC-ROC) for several prediction methods and the median AUC-ROC for evoKGsim using the best SSM.

**Table 5:** Summary of AUC-ROC with several PPI predicton methods, including evoKGsim methodology

Dataset	evoKGsim	ULCA by [13]	ULCA by [11]	WULCA by [11]	WAA by [11]	Best SSM by [17]
STRING-SC	0.89	0.83	0.92	0.95	0.95	
STRING-HS	0.94	0.85	0.90	0.93	0.95	
STRING-EC	0.92	0.93	0.93	0.96	0.96	
STRING-DM	0.98	0.82	0.82	0.86	0.85	
DIP-HS	0.97					0.92
BIND-SC	0.97			0.96	0.94	
DIP/MIPS-SC	0.87			0.93	0.93	
GRID/HPRD-bal-HS	0.74			0.68	0.67	
GRID/HPRD-unbal-HS	0.80			0.83	0.82	

The results in the third to sixth columns are all based on a similar approach, whereby an interacting protein pair is described by a vector that combines the presence/absence of GO terms for both proteins. The ULCA (up to lowest common ancestors) variant takes all annotations, direct and inherited up to the lowest common ancestor. The AA variant takes all annotations, direct and inherited. The weighted variants (WULCA and WAA) weight the presence of a GO term by its information content (IC). This is not a semantic-similarity based approach, but rather a propositional feature vector approach over the GO KG. The third column shows the best prediction performance of the ULCA with a Naïve Bayes classifier using the BP aspect obtained by Maetschke et al. [13]. The fourth, fifth, sixth columns present the results obtained by cross-validation of SVM otained by Bandyopadhyay and Mallick using all aspects [11]. The seventh column refers to an improved algorithm proposed by [13] to compute SS between GO terms annotated to proteins in benchmark interaction datasets.

Bandyopadhyay and Mallick [11] is the most recent work where the impact of the GO KG updates introduces less bias in a comparison with our results. An important difference between Bandyopadhyay and Mallick’s approach and ours, is that while ours uses semantic similarity as the features characterizing a protein pair, they employ IC weighted vectors of the GO terms assigned to each protein. Their approach gives the machine learning algorithm access to the annotations themselves, with models being able to learn exactly which annotations are better interaction predictors, while in evoKGsim the model is only able to learn which semantic aspects are the best predictors.

The Onto2Vec method, proposed by Smaili et al. [12], is also applied to predict PPIs in human and yeast. Although they did not use our benchmark datasets, PPIs were collected from STRING, the same database of PPIs from STRING-SC and STRING-HS datasets. In this work, Onto2Vec was used to learn feature vectors for proteins combining information about their GO annotations and the semantics of the GO terms in a single representation. The best AUC-ROC values were 0.8869 and 0.8931 for yeast and human datasets, respectively, and were obtained using an artificial neural network on the Onto2Vec representations.

## Conclusions

Knowledge-graph based semantic similarity measures have several very important biomedical applications, ranging from the prediction of protein-protein interactions, of gene product function or even of genes associated with diseases. Using KG-based SSMs typically includes selecting the aspects of the KG that are relevant for a given target application, a task that needs expert knowledge.

We have developed a novel approach, evoKGsim, that is able to learn suitable combinations of SS aspects to support supervised learning using GP. We evaluated its performance in protein-protein interaction prediction using the Gene Ontology as the KG (with its three semantic aspects: molecular function, biological process and cellular component) and a set of nine benchmark datasets.

evoKGsim is able to learn suitable combinations of SS aspects that improve PPI prediction performance over classical static combinations and classical classification algorithms like decision trees. The results have also revealed that exhaustive-like searches can provide comparable results to our methodology, but at the cost of increased computational effort. To overcome the limitation imposed by smaller datasets, we have also demonstrated that a model trained on one or multiple other species can be transferred and successfully be applied to a different species.

There are several avenues for future work, including the application to different supervised learning tasks, adding more SSMs to the evaluation, and combining our approach for semantic aspect selection with the more recent approaches based on graph embeddings. Despite the narrow application proposed here, evoKGsim can also be generalized to other applications and domains, such as disease gene discovery and prioritization using the Human Phenotype Ontology, or link prediction over KGs.

## Methods

An overview of the evoKGsim methodology is shown in Fig. 10. In a first step, the semantic similarities corresponding to each semantic aspect are computed for each protein pair in our input data. In a second step, GP evolves a good (hopefully the best) combination of the different SS aspects to support PPI prediction. Finally, the quality of the classifications obtained on the test set, using the evolved combination, is evaluated.

The implementation of our methodology takes as input an ontology file, a protein annotation file and a list of protein pairs. The Semantic Measures Library 0.9.1 [44] is used to compute the SSMs using GO and GO annotations. Two machine learning and GP libraries are used in the second step: scikit-learn 0.20.2 [34] and gplearn 3.0 (https://gplearn.readthedocs.io).

### Data sources

Data sources are organized in KG and benchmark datasets, which are described in the next subsections.

#### Knowledge graph

The KG used in this work is composed by the GO and GO annotations. GO [5] (dated January 2019) contains 45006 ontology terms subdivided into 4206 cellular component terms, 29689 biological process terms, and 11111 molecular function terms. Only *is-a* relations are considered. GO annotations are downloaded from Gene Ontology Annotation (GOA) database [45] (dated January 2019) for different species. These link Uniprot identifiers for proteins with GO terms describing them.

GO [5] is the most widely-used biological ontology. GO defines the universe of concepts (also called “GO terms”) associated with gene product^1^ functions and how these functions are related with each other with respect to three aspects: (i) biological process (BP), which captures the larger process accomplished by multiple molecular activities in which the gene product is active; (ii) molecular function (MF), biochemical (or molecular-level) activity of a gene product; (iii) cellular component (CC), the location relative to cellular structures in which a gene product performs a function. GO terms and their semantic relations form a hierarchical directed acyclic graph (DAG) where the three GO aspects are represented as root nodes of the graph. The ancestor terms in the hierarchy subsume the semantics of descendent terms.

A GO annotation associates a specific gene product with a specific term in the GO, identifying some aspect of its function. For instance, in Fig. 1 the gene product for *ACES HUMAN* is annotated with the GO term *amyloid percursor protein metabolic process*. A single gene product may be annotated with several terms across all semantic aspects of GO.

#### Benchmark protein-protein interaction datasets

For evaluation and comparison, we use benchmark PPI datasets of different species. These datasets were produced by other works and have been applied by several others in evaluating PPI approaches (see Table 6). The positive data (interacting protein pairs) of these datasets were collected from existing databases. The negative data is obtained by random sampling of protein pairs, since experimental high-quality negative data (non-interacting protein pairs) is hardly available. Random sampling is based on the assumption that the expected number of negatives is several orders of magnitude higher than the number of positives, such that the negative space is randomly sampled with larger probability than the positive space [43]. In most of the datasets, negative data is generated by randomly creating protein pairs that are not reported to interact. In the dataset GRID/HPRD-bal-HS a different strategy is employed to achieve balanced random sampling. Here, the number of times each protein appears in the negative set is equal to the number of times it appears in the positive set, with the negative set still being composed of protein pairs that are not known to interact.

**Table 6:** PPI benchmark datasets, with number of positive interactions (PI) and number of negative interactions (NI)

Dataset	Species	PI	NI
STRING-SC [13]	*S. cerevisiae*	15218	15166
STRING-HS [13]	*H. sapiens*	3460	3452
STRING-EC [13]	*E. coli*	1127	1118
STRING-DM [13]	*D. melanogaster*	288	262
DIP-HS [17]	*H. sapiens*	1375	1364
BIND-SC [37]	*S. cerevisiae*	724	642
DIP/MIPS-SC [37]	*S. cerevisiae*	4659	9148
GRID/HPRD-bal-HS [38]	*H. sapiens*	15675	15674
GRID/HPRD-unbal-HS [38]	*H. sapiens*	15675	15645

The species and the number of interactions for each dataset are provided in Table 4. Given the evolving nature of GO annotations, some benchmark proteins are no longer found in current GOA files. Consequently, we removed all pairs that failed to meet this criterion: both proteins have at least one annotation in one semantic aspect. Furthermore, the yeast datasets do not use Uniprot identifiers. We used the Protein Identifier Cross-Reference (PICR) tool [46] web application to map protein identifiers to the corresponding UniProt accession numbers. PICR provides programmatic access through Representational State Transfer (REST) that is very useful since we simply need to build a well-formatted RESTful URL. Thus, not all identifiers could be mapped to Uniprot and those proteins were removed.

Table S1 of Additional file 1 provides the number of interactions for each dataset before excluding the pairs that did not meet the above criteria.

### Semantic similarity measures

A SSM is a function that, given two ontology terms or two sets of terms annotating two entities, returns a numerical value reflecting the closeness in meaning between them. Thus, SS can be calculated for two ontology terms, for instance calculating the similarity between the GO terms *protein metabolic process* and *protein stabilization*; or between two entities each annotated with a set of terms, for instance calculating the similarity between *APBB1 HUMAN* and *ACES HUMAN*. In the case of proteins annotated with GO, SS can be interpreted as a measure of functional similarity between proteins.

Many SSMs applied to biomedical ontologies have been proposed, see for instance [14, 47, 48] and references therein. Early approaches for term semantic similarity have used path distances between terms, assuming that all the semantic links have equal weight. More recent approaches explore the notion of information content (IC), a measure of how specific and informative a term is. This gives SSMs the ability to weight the similarity of two terms according to their specificity. IC can be calculated based on intrinsic properties, such as the structure of the ontology, or using external data, such as the frequency of annotations of entities in a corpus. Taking Fig. 1 as an example, this allows SSMs to consider *protein catabolic process* and *amyloid precursor protein metabolic process* more similar than *protein metabolic process* and *protein stabilization*.

Entity SSMs typically employ one of two approaches: (1) pairwise: where pairwise comparisons between all terms annotating each entity are considered; (2) groupwise: where set, vector or graph-based measures are employed, circumventing the need for pairwise comparisons. Figure 11 illustrates how two proteins are represented by their GO terms when some terms annotate only one protein while others annotate both proteins.

In this work, the SS between two proteins is computed using three different SSMs (SimGIC, *Resnik*_*Max*_ and *Resnik*_*BMA*_), summarized in Table 7. SimGIC is a groupwise approach proposed by Pesquita et al. [49], based on a Jaccard index in which each GO term is weighted by its IC and given by

1 $$ \text{simGIC}(p_{1},p_{2}) = \frac{ \sum_{t \in \{\text{GO}(p_{1}) \cap \text{GO}(p_{2})\}}\text{IC}(t)}{ \sum_{t \in \{\text{GO}(p_{1}) \cup \text{GO}(p_{2})\}}\text{IC}(t)}   $$

**Table 7:** Summary of SSMs used to calculate the SS between gene-products

SSM	IC	Approach	Measure
SimGIC	Intrinsic	graph-based	Jaccard
Resnik_Max_	Intrinsic	best pairs	Maximum IC
Resnik_BMA_	Intrinsic	best pairs	Average IC

where GO(*p*_*i*_) is the set of annotations (direct and inherited) for protein *p*_*i*_.

*Resnik*_*Max*_ and *Resnik*_*BMA*_ are pairwise approaches based on the term-based measure proposed by Resnik [50] in which the similarity between two terms corresponds to the IC of their most informative common ancestor. This pairwise approach is used with two combination variants, maximum

2 $$ \begin{aligned} &\text{Resnik}_{\text{Max}}(p_{1},p_{2}) = \\ &\hspace{5mm}\max{\{\text{sim}(t_{1},t_{2}): t_{1} \in \text{GO}(p_{1}), t_{2} \in \text{GO}(p_{2})\}} \end{aligned}   $$

and best-match average

3 $$ \begin{aligned} \text{Resnik}_{\text{BMA}}(p_{1},p_{2}) = & \frac{\sum_{t_{1} \in \text{GO}(p_{1})}\text{sim}(t_{1},t_{2})}{2|{\text{GO}(p_{1})}|} + \\ & \frac{\sum_{t_{2} \in \text{GO}(p_{2})}\text{sim}(t_{1},t_{2})}{2|{\text{GO}(p_{2})}|} \end{aligned}   $$

where |GO(*p*_*i*_)| is the number of annotations for protein *p*_*i*_ and sim(*t*_1_,*t*_2_) is the SS between the GO term *t*_1_ and GO term *t*_2_ and is defined as

4 $$ \text{sim}(t_{1},t_{2})= \max{\{ \text{IC}(t) : t \in \{\mathrm{A}(t_{1}) \cap \mathrm{A}(t_{2})\}\}}   $$

where A(*t*_*i*_) is the set of ancestors of *t*_*i*_.

These measures were selected because *SimGIC* and *Resnik*_*BMA*_ represent high-performing group and pairwise approaches in predicting sequence, Pfam and Enzyme Commission similarity [49], whereas *Resnik*_*Max*_ may help elucidating whether a single source of similarity is enough to establish interaction.

The IC of each GO term is calculated using a structure-based approach proposed by Seco et al. [51] based on the number of direct and indirect descendants and given by

5 $$ \text{IC}_{\text{Seco}}(t) = 1 - \frac{\log{\bigl[\text{hypo}(t)+1\bigr]}\, }{\log{\bigl[\text{maxnodes}\bigr]}\,}   $$

where hypo(*t*) is the number of direct and indirect descendants from term *t* (including term *t*) and maxnodes is the total number of concepts in the ontology.

### Genetic programming and supervised learning

GP [33] is one of the methods of evolutionary computation [52–54] that is capable of solving complex problems by evolving populations of computer programs, using Darwinian evolution and Mendelian genetics as inspiration. GP can be applied to supervised learning problems [33, 55], including several in the biomedical domain (e.g. [56–58]).

**Fig. 12:**
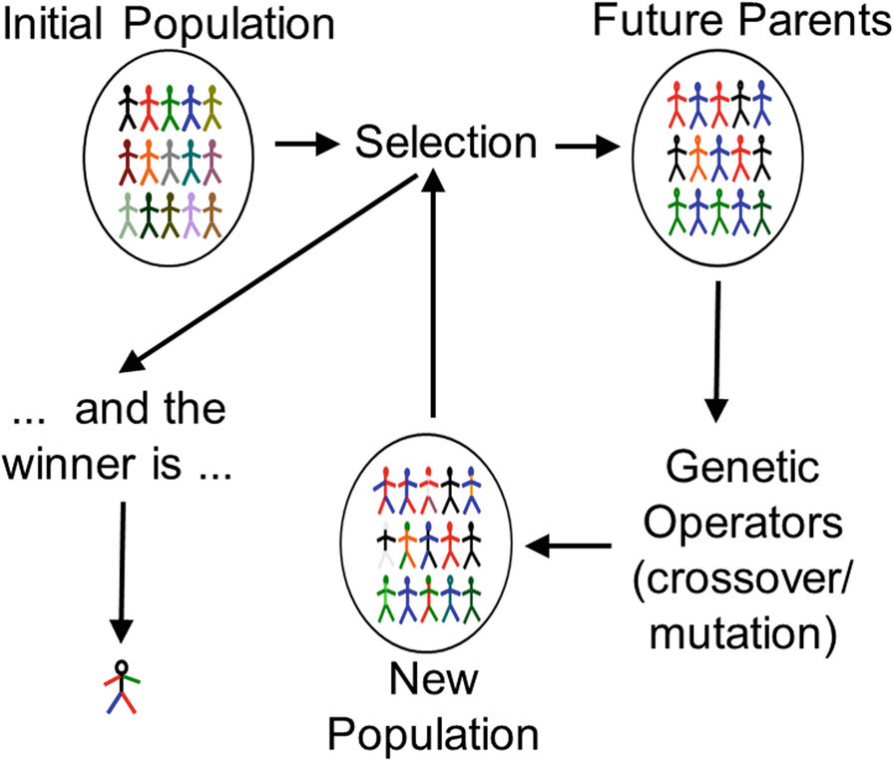
Genetic Programming Flowchart

Figure 12 illustrates the basic GP evolutionary cycle. Starting from an initial population of randomly created programs/models representing the potential solutions to a given problem (e.g., combinations of SS aspects to predict PPI), it evaluates and attributes a fitness value to each of them, quantifying how well the program/model solves the problem (e.g., what is the F-measure obtained). New generations of programs are iteratively created by selecting parents based on their fitness and breeding them using (independently applied) genetic operators like crossover (swapping of randomly chosen parts between two parents, thus creating two offspring) and mutation (modification of a randomly chosen part of a parent, thus creating one offspring). The fitter individuals are selected more often to pass their characteristics to their offspring, so the population tends to improve in quality along successive generations. This evolutionary process continues until a given stop condition is verified (e.g, maximum number of generations, or fitness reaching some threshold), after which the individual with the best fitness is returned as the best model found.

**Fig. 13:**
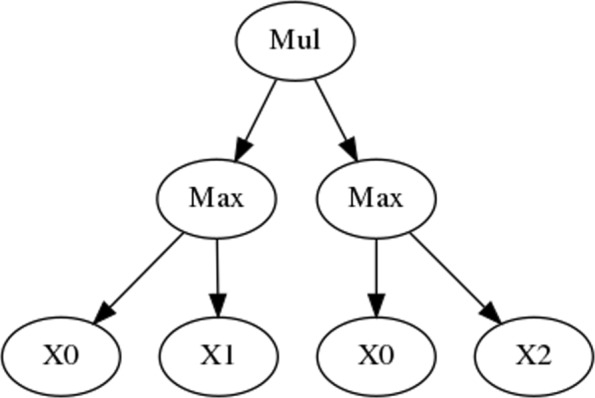
Example of a combination generated by GP. Variables X0, X1 and X2 represent the SS for BP, CC, and MF, respectively. Mul stands for Multiplication, and Max stands for Maximum

Theoretically, GP can solve any problem whose candidate solutions can be measured and compared. It normally evolves solutions that are competitive with the ones developed by humans [59], and sometimes surprisingly creative. GP implicitly performs automatic feature selection, as selection promptly discards the unfit individuals, keeping only the ones that supposedly contain the features that warrant a good fitness. Unlike other powerful machine learning methods (e.g., Deep Learning), GP produces ’white-box’ models, potentially readable depending on their size. For PPI prediction, the models evolved by GP are simply combinations of the SS of the three semantic aspects. In tree-based GP (the most common type), these models are represented as parse trees that are readily translated to readable strings. Figure 13 shows a parse tree of one of the simplest combinations evolved in our experiments, here translated as

6 $$ \max{(BP,CC)} \times \max{(BP,MF)}   $$

where the SS aspects BP, CC and MF are the variables *X*0, *X*1, and *X*2, respectively. These three variables constitute what is called the terminal set in GP, as they are only admitted as terminal nodes of the trees. In contrast, the function set contains the operators that can be used to combine the variables, and can only appear in internal nodes of the trees. The function set is a crucial element in GP. Together with the fitness function and the genetic operators, it determines the size and shape of the search space.

Given the free-form nature of the models evolved by GP, its intrinsic stochasticity, and the size of the search space where it normally operates, there is high variability among the raw models returned in different runs, even when using the same settings and same dataset. Even upon simplification, these models normally remain structurally very different from each other, while possibly exhibiting similar behavior, i.e., returning similar predictions. This characteristic raises some difficulty in interpreting the GP models, even if they are fully readable. Either way, it is always advisable to run GP more than once for the same problem, to avoid the risk of adopting a sub-optimal model that may have resulted from a less successful search on such a large space.

We have used a “vanilla” tree-based GP system, with no extras to boost the performance. The parameters we have set are listed in Table 8. All others were used with the default values of the gplearn software and are listed in Table S2 of Additional file 1. The parsimony coefficient is a non-standard parameter, specific to gplearn, and consists of a constant that penalizes large programs by adjusting their fitness to be less favorable for selection. It was set to 10^−5^, a value experimentally found to reduce the size of the evolved models without compromising their fitness. The function set contained only the four basic arithmetic operators (+,−,×, and ÷, protected against division by zero as in [60]), plus the Maximum (max) and Minimum (min) operators. Although there is a vast array of tunable parameters even in the most basic GP system, normally they do not substantially influence the outcome in terms of best fitness achieved [61].

**Table 8:** GP parameters

Parameter	Value
Number of generations	50
Size of population	500
Function set	+,−,×,÷,max,min
Fitness function	RMSE
Parsimony coefficient	10^−5^

For binary classification, it is fairly standard to use GP in a regression-like fashion, where the expected class labels are treated as numeric expected outputs (0 for no interaction; 1 for interaction), and the fitness function that guides the evolution is based on the error between the expected and predicted values [62]. We have used this same system in our experiments, with the Root Mean Squared Error (RMSE) as fitness function [63]. However, when we report the performance of evoKGsim, we first transform the real-valued predicted outputs in class labels, by applying the natural cutoff of 0.5.

### Performance measures

The classification quality is evaluated using the weighted average of F-measures (WAF). This metric accounts for class unbalance by computing the F-measure for each class and then calculating the average of all computed F-measures, weighted by the number of instances of each class:

7 $$ \text{WAF} = \frac{\sum_{c \in C} \text{F-measure}_{\text{c}} \times \text{Support}_{\text{c}}}{\sum_{c \in C}\text{Support}_{\text{c}}}   $$

where *C* is the set of classes, F-measure_*c*_ is the F-measure computed for class *c*, and Support_*c*_ is the number of instances in class *c*.

In each experiment, we perform stratified 10-fold cross-validation. The same folds are used throughout all experiments. At the end of each fold, we evaluate the WAF of classifications on the respective test set and report the median.

## Supplementary information

**Additional file 1** Supplementary figures and tables.

## Data Availability

All data generated and/or analyzed during this study are included in this published article and its supplementary information file.

## Abbreviations

AUC-ROC: area under the receiver operating characteristic curve
BP: biological process
CC: cellular component
GO: gene ontology
GOA: gene ontology annotation
GP: genetic programming
IC: information content
KG: knowledge graph
MF: molecular function
PICR: protein identifier cross-reference
PPI: protein-protein interaction
RDF: resource description framework
REST: representational state transfer
RMSE: root mean square error
SS: semantic similarity
SSM: semantic similarity measure
SVM: support vector machine
ULCA: up to lowest common ancestor
WAA: weighted all terms
WAF: weighted average F-measure
WULCA: weighted up to lowest common ancestor

## Footnotes

1 proteins or RNA
