# Supplementary material for: Evolving knowledge graph similarity for supervised learning in complex biomedical domains
Source: BMC Bioinformatics. 2020 Jan 3;21:6. doi: 10.1186/s12859-019-3296-1 (PMC6942314; doi:10.1186/s12859-019-3296-1)
Supplement: Supplementary file 1 — Additional file 1 Supplementary figures and tables. [file 12859_2019_3296_MOESM1_ESM.pdf]

Evolving knowledge graph similarity for supervised learning in complex biomedical domains (Supplementary material)

Rita T Sousa*, Sara Silva and Catia Pesquita

*Correspondence: risousa@ciencias.ulisboa.pt
LASIGE, Faculdade de Ciências, Universidade de Lisboa, Lisboa, Portugal
Full list of author information is available at the end of the article

Table S1 Original PPI Benchmark Datasets with their numbers of total interactions (I), positive interactions (PI) and negative interactions (NI). The STRING-SC, STRING-HS, STRING-EC, STRING-DM datasets are available in http://bioinformatics.org.au/tools/go2ppi/#training. The DIP-HS is avaliable in http://baderlab.org/Software/TCSS. The BIND-SC and DIP/MIPS-SC are available in https://noble.gs.washington.edu/proj/sppi/. The GRID/HPRD-bal-HS and GRID/HPRD-unbal-HS are available in http://www.bioinformatics.leeds.ac.uk/BRS-nonint/PPI_RandomBalance.html.

Dataset	I	PI	NI
STRING-SC	30476	15238	15238
STRING-HS	6980	3490	3490
STRING-EC	2334	1167	1167
STRING-DM	642	321	321
DIP-HS	2826	1391	1435
BIND-SC	1499	749	750
DIP/MIPS-SC	14498	4825	9673
GRID/HPRD-bal-HS	31608	15804	15804
GRID/HPRD-unbal-HS	31608	15804	15804

Table S2 Default GP parameters.

Parameters	Value
Tournament size	20
Stopping criteria	0.0
Range of constants to include in formulas	[-1.0,1.0]
Range of tree depths for the initial population	[2,6]
Initialization method	ramped half and half
Probability of crossover on a tournament winner	0.9
Probability of subtree mutation on a tournament winner	0.01
Probability of hoist mutation on a tournament winner	0.01
Probability of point mutation on a tournament winner	0.01
Probability of any given node will be mutated, for point mutation	0.05

Table S3 p-values for the Kruskal-Wallis test for comparisons between the evolved combinations and all other baselines over the nine PPI datasets.

Dataset	SSM	BP vs EC	CC vs EC	MF vs EC	Avg vs EC	Max vs EC	Exhaustive Search vs EC	Decision Tree vs EC
STRING-EC	SimGIC	0.00016	0.32575	0.00016	0.44969	0.05878	0.82060	0.17362
	$\text{Resnik}_{\text{Max}}$	0.00016	0.00115	0.00016	0.00150	0.00650	0.00150	0.36435
	$\text{Resnik}_{\text{BMA}}$	0.00016	0.01017	0.00016	0.05878	0.38449	0.04125	0.38378
STRING-DM	SimGIC	0.14002	0.08198	0.00028	0.27195	0.18555	0.76167	0.01717
	$\text{Resnik}_{\text{Max}}$	0.24061	0.01248	0.00016	0.84949	0.47167	0.34325	0.21143
	$\text{Resnik}_{\text{BMA}}$	0.08849	0.00043	0.00015	0.51926	0.05850	0.54368	0.08106
BIND-SC	SimGIC	0.13043	0.00514	0.00021	0.24114	0.04928	0.59670	0.03429
	$\text{Resnik}_{\text{Max}}$	0.05869	0.00406	0.00016	0.52037	0.22630	0.67747	0.49629
	$\text{Resnik}_{\text{BMA}}$	0.09630	0.00149	0.00029	0.59642	0.02569	0.54535	0.02837
DIP/MIPS-SC	SimGIC	0.04937	0.00016	0.00016	0.00815	0.00029	0.11241	0.00016
	$\text{Resnik}_{\text{Max}}$	0.22648	0.00016	0.00016	0.02334	0.01017	0.65015	0.08210
	$\text{Resnik}_{\text{BMA}}$	0.00815	0.00016	0.00016	0.49629	0.00516	0.93974	0.00016
STRING-SC	SimGIC	0.00067	0.00016	0.00016	0.00150	0.00016	0.32575	0.00016
	$\text{Resnik}_{\text{Max}}$	0.00067	0.00016	0.00016	0.01017	0.00038	0.19876	0.70546
	$\text{Resnik}_{\text{BMA}}$	0.00016	0.00016	0.00016	0.36435	0.00016	0.44969	0.00016
DIP-HS	SimGIC	0.19876	0.00016	0.00016	0.00194	0.00016	0.62305	0.00016
	$\text{Resnik}_{\text{Max}}$	0.76237	0.00250	0.00021	0.25684	0.05878	0.36435	0.38449
	$\text{Resnik}_{\text{BMA}}$	0.40568	0.00021	0.00021	0.19876	0.00029	0.40568	0.08210
STRING-HS	SimGIC	0.09630	0.00016	0.00016	0.00650	0.00016	0.22648	0.00016
	$\text{Resnik}_{\text{Max}}$	0.00194	0.00016	0.00016	0.00650	0.00016	0.36399	0.00194
	$\text{Resnik}_{\text{BMA}}$	0.00115	0.00016	0.00016	0.01258	0.00016	0.17346	0.00016
GRID/HPRD-unbal-HS	SimGIC	0.28992	0.00016	0.00021	0.25684	0.00067	0.65015	0.00016
	$\text{Resnik}_{\text{Max}}$	0.22648	0.00115	0.00250	0.59670	0.02334	1.00000	0.04125
	$\text{Resnik}_{\text{BMA}}$	0.05878	0.00016	0.00051	0.54535	0.00088	0.93974	0.00016
GRID/HPRD-bal-HS	SimGIC	0.00115	0.00016	0.00016	0.40568	0.00029	0.76237	0.00016
	$\text{Resnik}_{\text{Max}}$	0.49629	0.00016	0.00038	0.19876	0.00320	0.70546	0.00150
	$\text{Resnik}_{\text{BMA}}$	0.00115	0.00016	0.00016	0.22648	0.00029	0.19876	0.00016

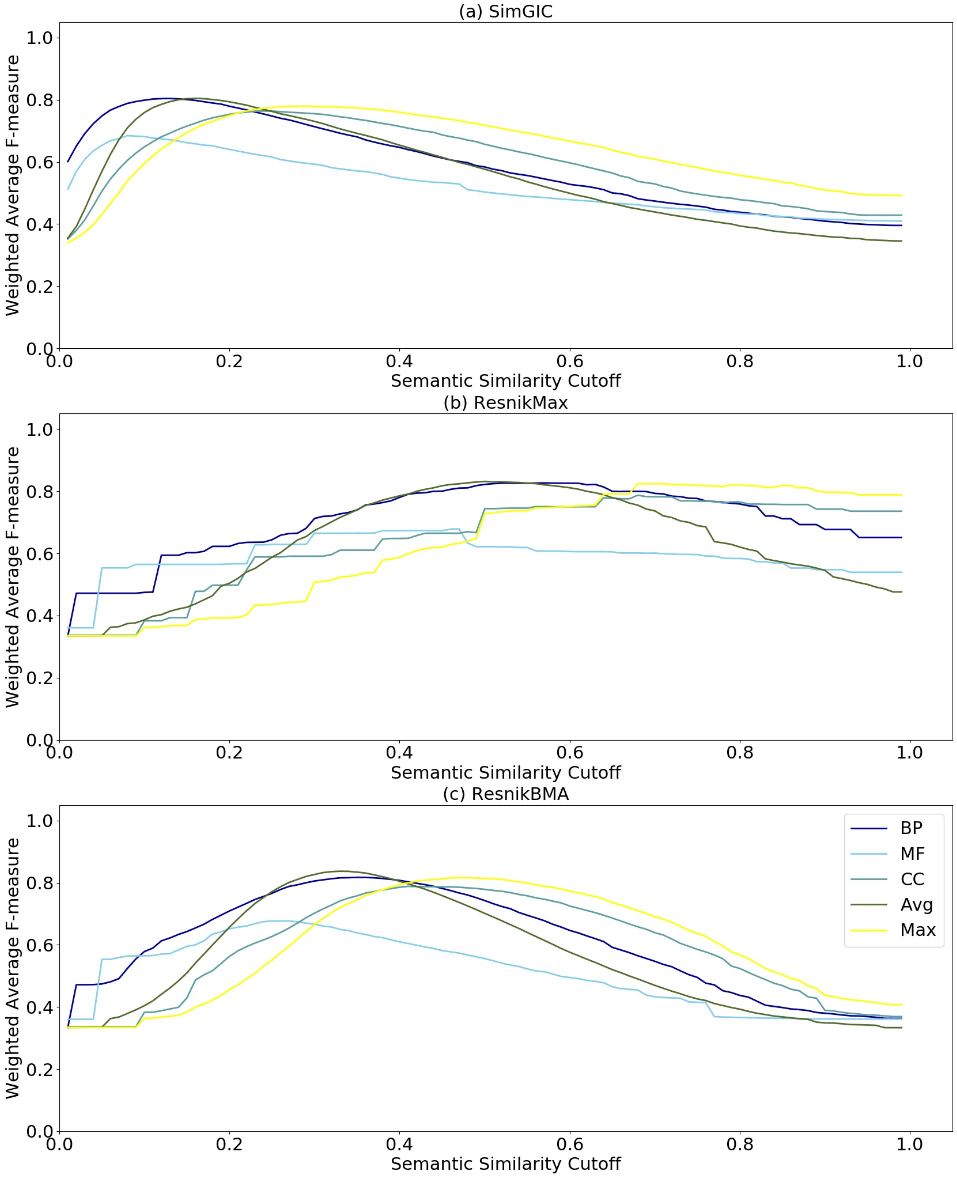

Figure S1 WAF Curves for STRING-SC PPI dataset. WAF evaluations with static combinations of semantic aspects (CC, BP, MF, Avg and Max) at different cutoffs are shown. The evaluation is performed using three SSMs: (a) SimGIC, (b) $\text{Resnik}_{\text{Max}}$ and (c) $\text{Resnik}_{\text{BMA}}$.

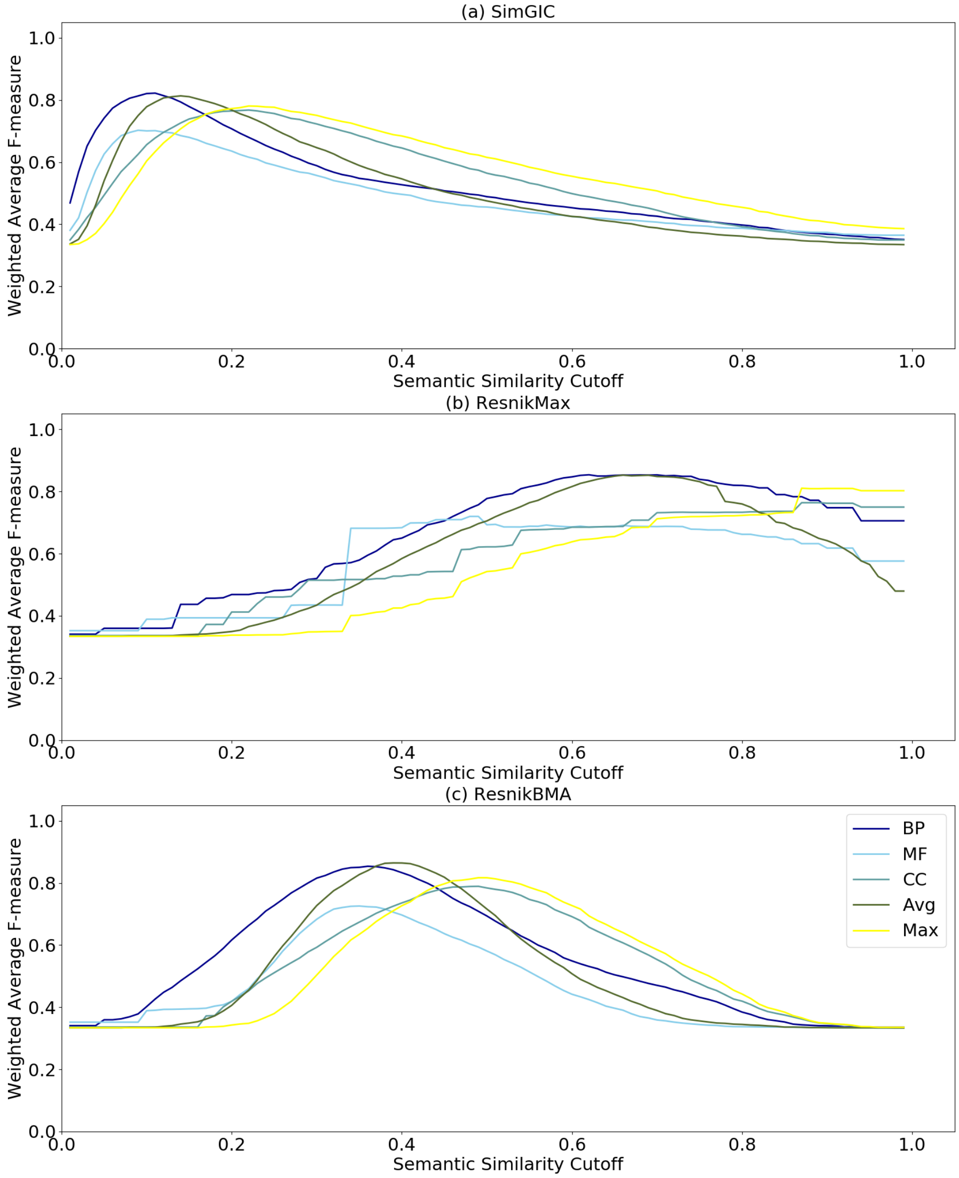

Figure S2 WAF Curves for STRING-HS PPI dataset. WAF evaluations with static combinations of semantic aspects (CC, BP, MF, Avg and Max) at different cutoffs are shown. The evaluation is performed using three SSMs: (a) SimGIC, (b) $\text{Resnik}_{\text{Max}}$ and (c) $\text{Resnik}_{\text{BMA}}$.

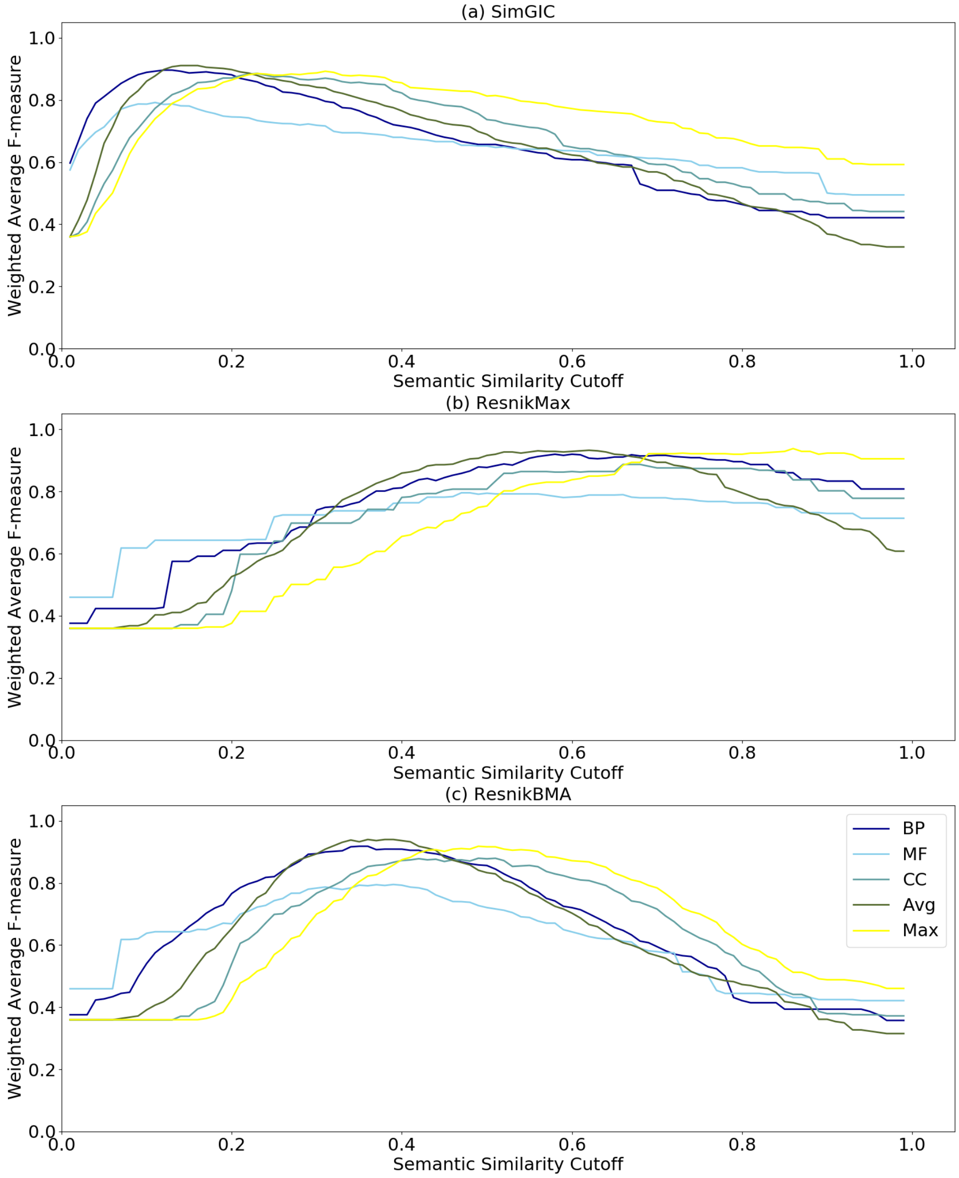

Figure S3 WAF Curves for STRING-DM PPI dataset. WAF evaluations with static combinations of semantic aspects (CC, BP, MF, Avg and Max) at different cutoffs are shown. The evaluation is performed using three SSMs: (a) SimGIC, (b) $\text{Resnik}_{\text{Max}}$ and (c) $\text{Resnik}_{\text{BMA}}$.

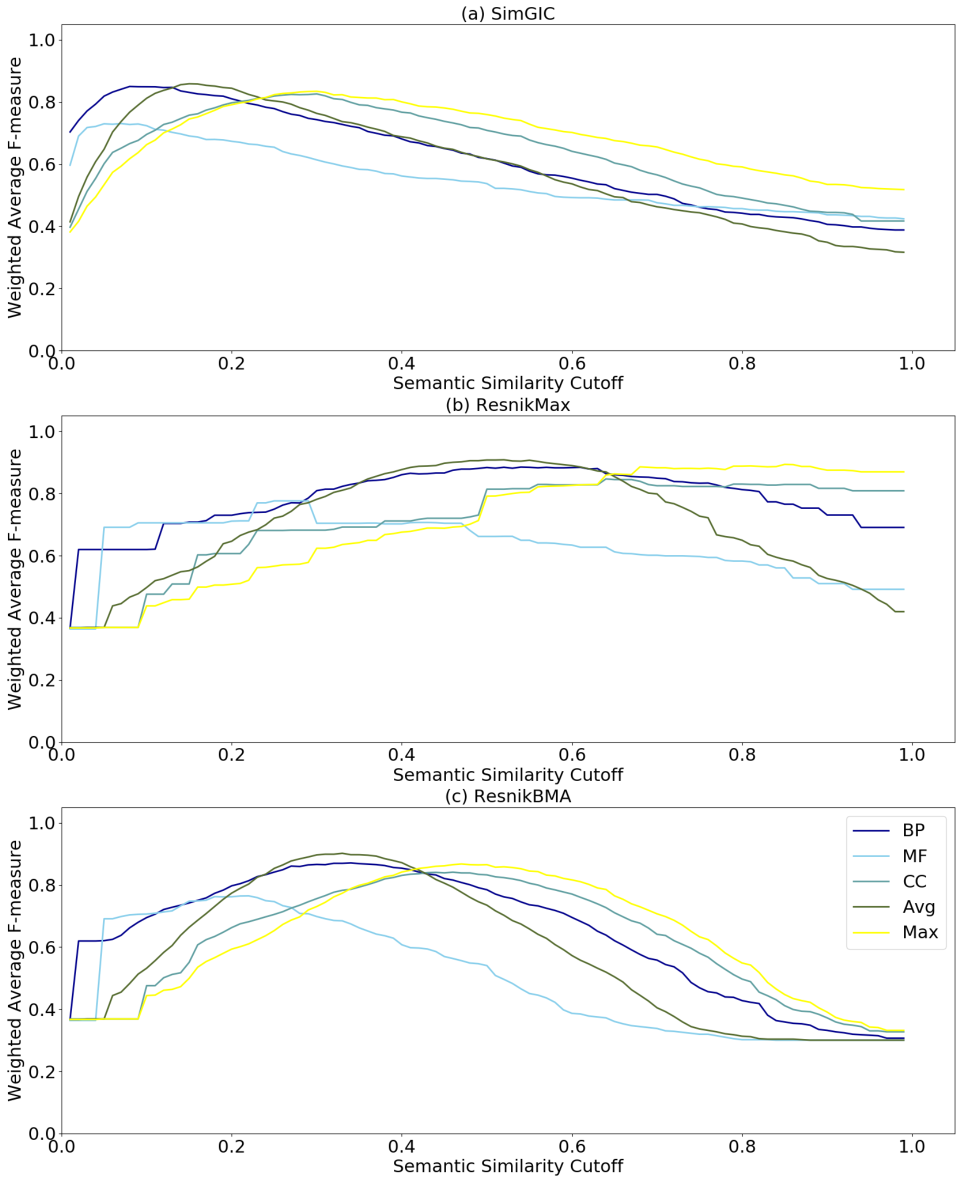

Figure S4 WAF Curves for BIND-SC PPI dataset. WAF evaluations with static combinations of semantic aspects (CC, BP, MF, Avg and Max) at different cutoffs are shown. The evaluation is performed using three SSMs: (a) SimGIC, (b) $\text{Resnik}_{\text{Max}}$ and (c) $\text{Resnik}_{\text{BMA}}$.

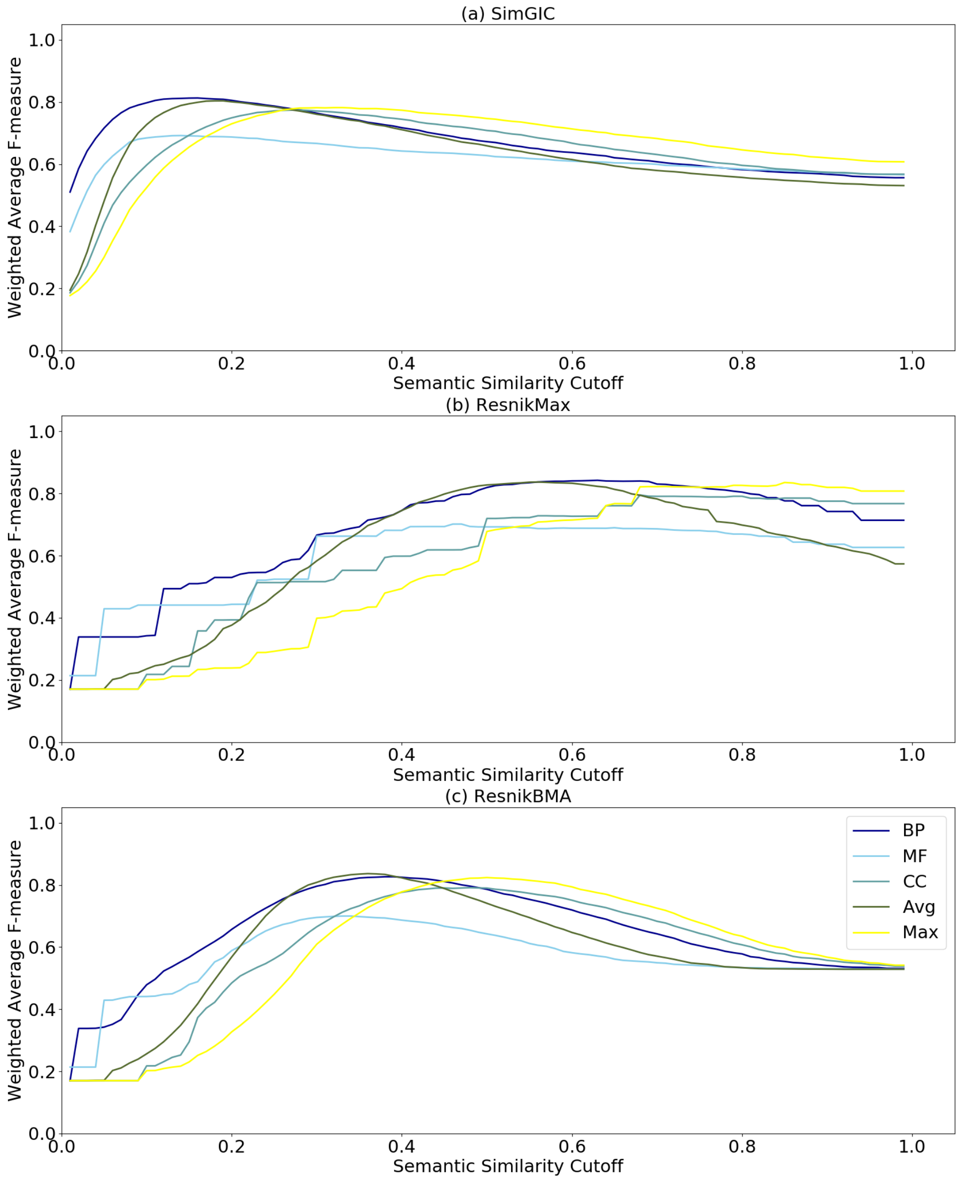

Figure S5 WAF Curves for DIP/MIPS-SC PPI dataset. WAF evaluations with static combinations of semantic aspects (CC, BP, MF, Avg and Max) at different cutoffs are shown. The evaluation is performed using three SSMs: (a) SimGIC, (b) $\text{Resnik}_{\text{Max}}$ and (c) $\text{Resnik}_{\text{BMA}}$.

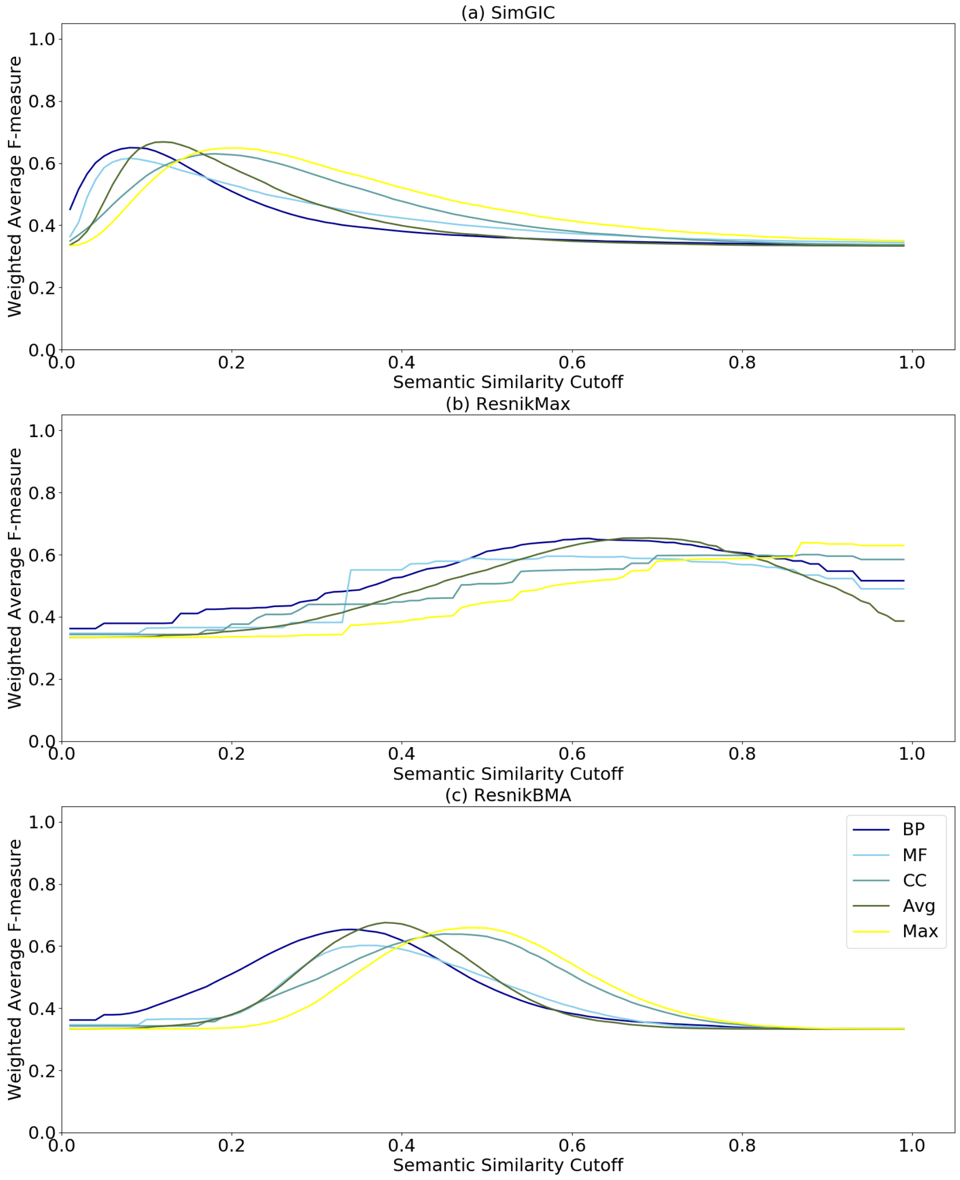

Figure S6 WAF Curves for GRID/HPRD-bal-HS PPI dataset. WAF evaluations with static combinations of semantic aspects (CC, BP, MF, Avg and Max) at different cutoffs are shown. The evaluation is performed using three SSMs: (a) SimGIC, (b) $\text{Resnik}_{\text{Max}}$ and (c) $\text{Resnik}_{\text{BMA}}$.

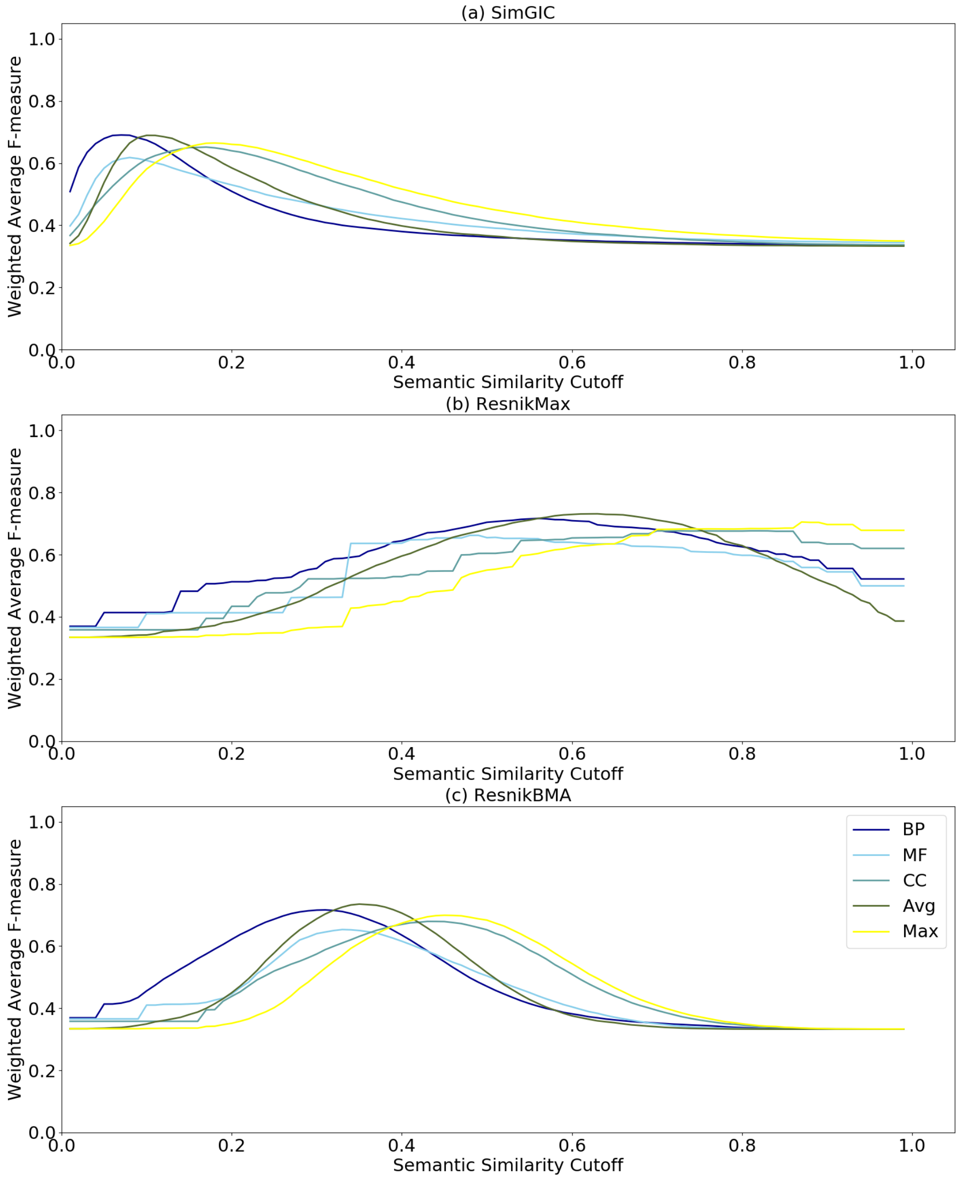

Figure S7 WAF Curves for GRID/HPRD-unbal-HS PPI dataset. WAF evaluations with static combinations of semantic aspects (CC, BP, MF, Avg and Max) at different cutoffs are shown. The evaluation is performed using three SSMs: (a) SimGIC, (b) $\text{Resnik}_{\text{Max}}$ and (c) $\text{Resnik}_{\text{BMA}}$.
